# Multiclass classification of environmental chemical stimuli from unbalanced plant electrophysiological data

**DOI:** 10.1371/journal.pone.0285321

**Published:** 2023-05-04

**Authors:** Nivedita Bhadra, Shre Kumar Chatterjee, Saptarshi Das

**Affiliations:** 1 Department of Physical Sciences, Indian Institute of Science Education and Research, Nadia, Kolkata, West Bengal, India; 2 Department of Electronics and Computer Science, University of Southampton, Southampton, United Kingdom; 3 Centre for Environmental Mathematics, Faculty of Environment, Science and Economy, University of Exeter, Exeter, United Kingdom; 4 Institute for Data Science and Artificial Intelligence, University of Exeter, Exeter, United Kingdom; University of Baghdad, IRAQ

## Abstract

Plant electrophysiological response contains useful signature of its environment and health which can be utilized using suitable statistical analysis for developing an inverse model to classify the stimulus applied to the plant. In this paper, we have presented a statistical analysis pipeline to tackle a multiclass environmental stimuli classification problem with unbalanced plant electrophysiological data. The objective here is to classify three different environmental chemical stimuli, using fifteen statistical features, extracted from the plant electrical signals and compare the performance of eight different classification algorithms. A comparison using reduced dimensional projection of the high dimensional features via principal component analysis (PCA) has also been presented. Since the experimental data is highly unbalanced due to varying length of the experiments, we employ a random under-sampling approach for the two majority classes to create an ensemble of confusion matrices to compare the classification performances. Along with this, three other multi-classification performance metrics commonly used for unbalanced data viz. balanced accuracy, F_1_-score and Matthews correlation coefficient have also been analyzed. From the stacked confusion matrices and the derived performance metrics, we choose the best feature-classifier setting in terms of the classification performances carried out in the original high dimensional vs. the reduced feature space, for this highly unbalanced multiclass problem of plant signal classification due to different chemical stress. Difference in the classification performances in the high vs. reduced dimensions are also quantified using the multivariate analysis of variance (MANOVA) hypothesis testing. Our findings have potential real-world applications in precision agriculture for exploring multiclass classification problems with highly unbalanced datasets, employing a combination of existing machine learning algorithms. This work also advances existing studies on environmental pollution level monitoring using plant electrophysiological data.

## 1. Introduction

Plants have an established electrical signal response in the form of action potential (AP), variation potential (VP) or local electric potential (LEP) [1], using which they monitor their surrounding environment and control their physiological functions through biochemical changes [2]. When such electrical signals or responses are analyzed, the conditions of the surrounding environment and the status of the physiology of the plant can be determined [3]. Previous research has shown, that by using 11 higher order statistical features computed from small windows of 1000 samples of the extracted *raw* plant electrical signals, sufficient information about the stimuli applied on the plants can be determined [3]. These electrical signals from the plants were extracted by conducting a series of experiments under laboratory conditions, applying Sodium Chloride (NaCl), Sulfuric Acid (H_2_SO_4_) and Ozone (O_3_) as stimuli on a variety of plants. The information about the applied stimuli were also obtained from small time windows, containing only 1024 samples, of *preprocessed* (optimal infinite impulse response or IIR filtered for drift removal) plant electrical signal, as described in Das *et al*. [4]. From each of these windows of filtered signals, fifteen statistical features were extracted which were then used for multiclass classification using customized decision trees. The optimal filtering criteria for drift removal in plant electrical signals, used in this work, was also established previously in [5]. In another research [6], the entire signal duration was considered for feature extraction (using the coefficients of fitted curves) in order to classify the applied chemical stimuli. All these previous works were fundamental in establishing that small windows or the entire duration of the plant electrical signal, obtained through carefully designed experiments, can indeed be utilized for classifying the applied chemical stimuli.

### 1.1 Background and motivation

As the domain of plant electrophysiological signal processing is still at a nascent stage, with no established guidelines on various aspects of the data processing pipeline, various researchers have applied different signal processing and machine learning techniques to analyze plant electrical responses. For example, plant electrical signal light-induced bioelectrogenesis was obtained by means of periodic illumination/darkness stimulation of broad bean (Vicia faba L.) leaves in [7]. Different types of electrical signals like action potentials, local electrical potentials, variation potential, system potential along with different kinds of electrodes are reviewed in [8], such as metal electrode, glass electrode, voltage and patch clamp, ion selective electrode etc. Another study [9] developed a non-invasive and high-throughput light-induced in situ method to record bioelectrical activity in plants. The authors reported stable periodic electrical potential changes in wheat leaves which were induced by light or darkness cycles. Classification of salt-tolerant and salt-sensitive cultivars were then carried out with the collected data. A new method for monitoring plant physiological processes is the photochemical reflectance indices which has been important in remote sensing of changes in photosynthetic processes under the action of stressors (excess light, changes in temperature, drought, etc.) as reported in [10, 11]. The effect of drought stress has been compared in time and frequency domain signal analysis for estimating physiological parameters like root vitality, maximal photochemical efficiency, relative amount of chlorophyll in [12]. Burning-induced changes in electrical signal [13] causes a change in leaf reflectance at broad spectral bands (400–500, 500–600, 600–700, and 700–800 nm) which has been reported in [14]. Investigation on the influence of electrical signals on difference reflectance indices calculated on the basis of these broad spectral bands and the analysis of connection of the indices with water content in plants has been reported in [15]. Recognition of action potential and non-action potential has been reported in [16], using waveform similarity in time and frequency domain. Comparison of different machine learning algorithms for plant signal classification has also been applied for discriminating different stress conditions like cold, low light, osmotic and no stress conditions in [17] and using spectral features in [18]. Time domain features extracted using empirical mode decomposition (EMD) algorithm was used in [19], to classify mineral deficiency in tomato plants like Calcium, Iron, Nitrogen, Manganese. Plant signal statistical feature based classification has been compared using the discriminant analysis and deep learning in [20], for five different stimuli including heat, wind, blue/red light and no stimuli. Protein and electrical signal are classified using graph neural networks and bidirectional long short term memory (LSTM) in [21]. Similarly, inverse modeling using system identification approach for light stimulus has been studied in [22]. Water stress in different intensities in the form of high and medium drought and its correlation with plant electrical response has been used for classification in olive trees [23]. Wavelet transform based analyses have also been reported on plant signals and photosynthetic activities in [24]. Salt stress effects as a binary classification was attempted using a convolutional neural network with generative adversarial network (GAN) data augmentation for performance boosting [25]. Benchmarking of classification algorithms for fruit herbivore induced biochemical stress has been reported in [26], using wide range of statistical features extracted from plant signals. These recent works mostly deal with monitoring the status of plant physiological processes by directly or indirectly using plant electrical signals excluding different chemical stress which is the main focus of this paper.

However, the goals of these previous works are different when compared to ours, as we have primarily focused on addressing a multiclass classification problem using an unbalanced dataset. Our goal in this work is to classify three different chemical stimuli (NaCl, H_2_SO_4_ and O_3_) using higher order statistical features extracted from different blocks of fixed number of 1024 samples of plant electrical signal responses, which were extracted from two different species of plants.

### 1.2 Novelty of this work

The focus of the current study is to solve an unbalanced multiclass classification problem, in order to monitor the environment by using plant electrical signals rather than studying plant electrophysiological processes. The current work is a significant extension of the related binary classification results [3] and decision tree based transformation of the multiclass problem into several binary classification problems [4], using a thorough benchmarking between multiple classifiers, performance metrics and statistical comparison. In this paper, we address the problem of multiclass classification when using an unbalanced dataset by exploring multiple existing multiclass classification algorithms and show that the discrimination results obtained are robust. We also report a comparison whether a dimensionality reduction before classification is helpful in this plant stimulus detection task. The ultimate objective is to increase the predictive accuracy of the classifiers to identify environmental chemical pollutants which has affected the plants.

The same 15 statistical features as reported in [4] were extracted from each block of the time series using non-overlapping sliding window which include multiple measures of descriptive statistics viz. mean (*μ*), variance (*σ*^2^), skewness (*γ*_*3*_ –third central moment), kurtosis (*κ*_*4*_ –fourth central moment), interquartile range (IQR). It also includes features such as Hjorth mobility, Hjorth complexity, detrended fluctuation analysis (DFA), Hurst exponent and wavelet packet entropy (*H*) which captures nonlinear, nonstationary and non-Gaussian behaviors. Frequency domain features such as average spectral power (*P*_*s*_), and higher order moments like the super-skewness (*γ*_*5*_ –fifth central moment) and super-flatness (*κ*_*6*_ –sixth central moment) [27, 28], Fano factor (*σ*^2^/*μ*) and correlation dimension (*d*_*c*_) have also been used [29]. Detailed descriptions of these features have already been discussed in [3, 4], although those works focused on simpler classifiers like discriminant analysis and decision trees. Moreover, these previous works involved splitting the multi-class classification problem into multiple binary classification problems and did not deal with the unbalanced datasets which the current paper mainly focusses on.

Additionally, we benchmark the multiclass classification performances using several other multiclass classifiers along with random under-sampling of the majority classes [30–32]. The concept of under-sampling of the majority classes for handling class imbalance has been shown to have less bias over the over-sampling of the minority classes using diverse approaches including evolutionary algorithms [33], weighted under-sampling [34], noise filtered scheme [35], clustering and instance selection approaches [36] and optimal hyper-parameter tuned classifier pipeline with multiple unbalanced classification performance metrics [37]. Using random Monte Carlo draws from the majority classes [38, 39], we then construct several confusion matrix ensembles for fixed feature set and classifier combination and report the distribution of the respective classification performances under a Monte Carlo cross-validation framework. Many machine learning studies have shown that the use of dimensionality reduction using PCA and similar family of statistical methods are likely to yield better classification accuracy as compared to the original high dimensional feature space [40, 41]. However, previous claims of PCA aided classification being always better than the original high dimensional feature space [42] are indeed problem specific [43] and depends on the choice of the classifier and feature combinations. In the current algorithmic benchmarking process, we found that these previous claims are valid for most of the classifiers. This conforms with other previous works where introducing PCA sometimes may yield inferior results [43–45]. This may be due to the fact that the underlying pattern in the data is highly nonlinear and may not be represented well using a linear feature transformation like the PCA. For our current highly unbalanced multi-class classification problem, we report a thorough benchmarking between different sets of classifier-feature pairs using the strongest principal components as the new hybrid feature vs. the original high dimensional features. The current work is a significant improvement over the existing classification pipelines using coefficients of fitted curves [6] and the approaches of splitting the multi-class problem into several binary classification problems and using balanced accuracy as a single classification performance evaluation metric [3, 4]. The robust analysis of the confusion matrices in the classification based on Monte Carlo under-sampling of the majority class and benchmarking on PCA vs. non-PCA (standard high dimensional) version is a new extension in the field of plant electro-physiological data classification approaches [46]. The current paper compares the results of multiclass unbalanced classification using three different performance metrics and their joint distributions both intuitively or qualitatively and also quantitatively through multivariate hypothesis testing.

Rest of the paper is organized as follows. Section 2 describes material and methods including plant electrical signal collection, feature extraction, unbalanced classification, description of classifier hyperparameters and performance metrics. Section describes the benchmarking of 10 classifiers in the high dimensional and reduced principal component feature space in terms of Monte Carlo sampling based performance distributions in order to carry out hypothesis testing to show significant difference in the classifier performance. Section 4 describes the summary of the achievements in this paper as compared to previous works along with future research in this direction, followed by the references.

## 2. Material and methods

In the following subsections we discuss the plant electrical signal acquisition under three types of chemical stress, followed by feature extraction and description of the highly unbalanced classification problem, outlier removal, feature correlation analysis and dimensionality reduction using principal component analysis. We next report the Monte Carlo under-sampling for handling unbalanced data along with hyper-parameter settings of the classification algorithms and robust classification performance metrics.

### 2.1 Electrical signals from plants under chemical stress, data collection and preprocessing

Previous studies in [3–6] have reported an exhaustive set of experimental data collection consisting of approximately ~38,000 instances. Out of these, approximately ~28,000 data instances (73% of the total data) were used for training and approximately ≈ 10,000 data instances (27% of the total data) were used for prospective or held out validation study i.e. independent testing of the trained classifier. The best classifier and feature combinations were selected by employing the stratified cross-validation [47, 48] on ∼73% of the total data in order to:

- avoid selecting an over-fitted model of the total data, and
- test the obtained best classifier-feature combinations on a section of independent data i.e. to estimate the performance of this combination in classifying completely unseen data.

The datasets used in this study, were from experiments conducted using three different stimuli—NaCl (applied to tomato plants), H_2_SO_4_ (applied to tomato and cabbage plants) and O_3_ (applied to tomato and cabbage plants) similar to [4]. A summary of the experimentally collected dataset is given in Table 1. Each plant was used only once for an experiment. Thus, every experiment had a fresh new plant of similar age, structure and growing conditions thereby nullifying the risk of any residual effects of the previous experiments corrupting the electrophysiological response of the plants.

**Table 1:** Experimental details for collecting plant electrophysiological response to three different stimuli.

Plant species used for experiments	Stimulus	Concentration of applied stimulus	Number of times stimulus applied per experiment	Sampling rate
Tomato and Cabbage	Sulfuric acid (H_2_SO_4_)	5 ml of 0.05/0.025M solution	Once	10 Hz
Tomato	Sodium chloride (NaCl)	5/10ml of 3M solution	Once	10 Hz
Tomato and Cabbage	Ozone (O_3_)	16 ppm/ 13.07 ppm	Multiple	10 Hz

For each plant, two stainless steel needle electrodes (from Bionen S.A.S) of 0.35 mm diameter and 15 mm length, were inserted around 5–7 mm into the top and middle of the plant stem. These electrodes, similar to those used in EMG, were inserted such that the sensitive active parts of the electrodes (2 mm) were in contact with the plant cells. A third electrode was inserted at the base of the plant for reference. These electrodes were then connected to an amplifier-Data Acquisition (DAQ) system [3–6] and the plants were put in a transparent plastic enclosure. This enclosure allowed the plants to be exposed to artificial light conditions provided by LED lights mimicking a day/night cycle of 12 hour each thereby responding to plant’s photosynthetic needs. As external light produces electrophysiological response in plants which can alter the response to specific stimulus, each experiment was conducted in a dark room. Also, the whole setup was protected from effects of electromagnetic interference by placement inside a Faraday cage, where a comparison between signals inside and outside the Faraday cage can be seen in [49, 50]. In order to avoid injury related response of the plant to corrupt the stimuli specific response, an approximate waiting period of 45 min after insertion of the electrodes was practiced. Electrical signals thus acquired by the electrodes were provided to a 2-channel high impedance (1015 W) electrometer (DUO 773, WPI, Sarasota, FL, USA) and the data was recorded through a 4-Channel DAQ (LabTrax, WPI) along with LabScribe software (WPI) which are detailed in [3–6, 22].

In this work, filtered plant signals have been used for feature extraction. The optimum infinite impulse response (IIR) filtering criteria has been reported in [5] where a high-pass filter was reportedly applied on the raw plant electrophysiological signal to eliminate the low frequency trends which left only the high frequency stochastic parts. This stochastic part of the signal was then divided into multiple windows of fixed sample sizes of 1024 samples. The 15 features were then extracted from each of these windows (but only from the post-stimulus parts of the signals) and were then normalized so that all features lie within a scale of 0–1 [4]. Use of the high-pass IIR filtering was required to remove the initial low frequency drift before the stimulus was applied. However, the pre-stimulus or rest condition has not been attempted to classify in this work since it has been tackled already in the previous study [5].

### 2.2 Unbalanced experimental data collected from plants under chemical stress

In this work, approximately ~38,000 data blocks as reported in [4], are used for training/testing the classifiers, with a focus on the data imbalance problem. Thus, although no held-out validation is used similar to the previous studies, here the training vs. testing data splitting were repeatedly done through a 50:50 split in a Monte Carlo randomization to eliminate class bias which is explained later. The experimental data blocks, consisting of 1024 samples each, were obtained from different experiments as explained in [4] with a sampling rate of 10 Hz. The number of blocks used for each stimulus are shown in Table 2, from which we can observe that the main imbalance in the dataset is due to the application of Ozone as a stimulus which contributed 94% of the overall dataset. This large number of data blocks for Ozone was due to the longer duration of the experimental recording, in comparison to the other two stimuli [3]. In an ideal scenario of cheap and easy experimental data collection, it is often argued to train the classifier on a portion of the available dataset and then testing the model on a completely different experimental data commonly known as the held-out validation. Whereas we here employ a Monte Carlo cross validation methodology [46] with the following steps:

- Select equal number of samples for all three classes (equal number is dependent on the least represented class, i.e. NaCl) to create a sub-sample dataset,
- Divide this sub-sample dataset equally into *train* and *test* datasets (i.e. 50:50 split),
- These datasets are then used for classification and the above two steps are repeated 1000 times, which seems to be sufficiently high to eliminate any bias due to non-inclusion of certain group of heterogeneous datapoints. The results for all the 1000 repetitions are then reported as summary statistics of the ensemble confusion matrices for comparing different classifiers.

**Fig 1:**
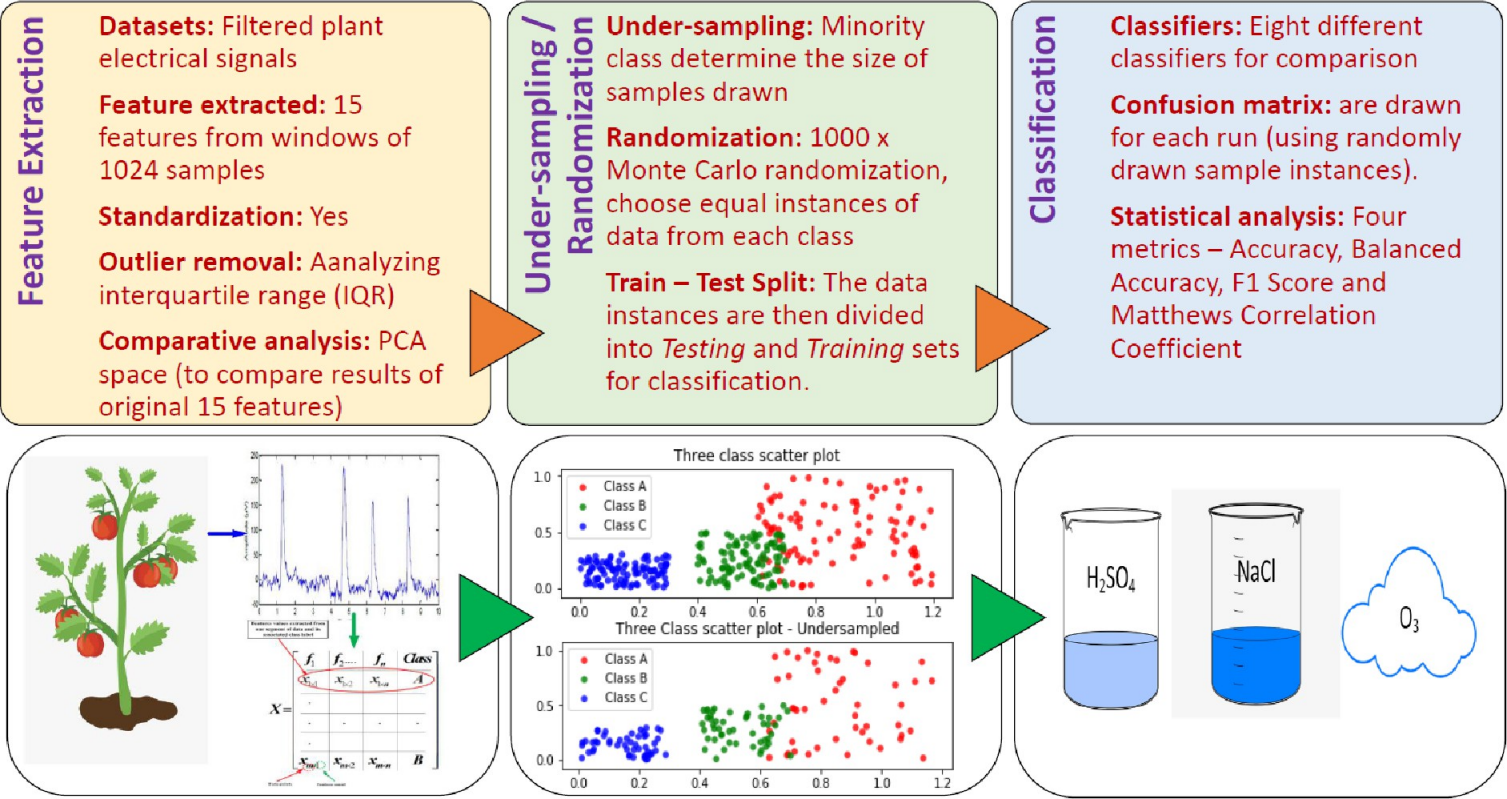
Schematic diagram of the plant signal data analysis pipeline with three basic steps.

The overall methodology is shown in Fig 1, starting from feature extraction from plant signals to Monte Carlo under-sampling followed by testing the performance of several classifiers.

**Table 2:** Data blocks (consisting of 1024 samples) belonging to each chemical stimulus.

NaCl	H_2_SO_4_	O_3_	Total
628	1488	35718	37834

Here, we do not explicitly adopt the usual notion of cross-validation due to high class-imbalance. Rather, we have used 1000 Monte Carlo runs of the random under-sampling which is sufficiently large number for ensemble statistics calculation with moderate computational burden. The 50:50 split of training and testing data under each draw of the Monte Carlo under-sampling scheme can be seen as an ensemble 2-fold cross validation. Using higher folds and the extreme case where the number of folds equals the number of datapoints–leave one out cross validation (LOOCV) are avoided due to massive increase in the computational burden. However, the datapoints from all the three classes are shuffled in the random draws of under-sampling scheme and can be viewed as a Monte Carlo cross-validation as described in [46]. In a block partitioning of a classification, other split percentages like 60:40, 70:30 or 80:20 could have been used instead for held out cross validation. However, under our cross-validation setting we randomly draw equal amount of data from each class which are then partitioned into a 50:50 split to avoid any potential bias for the samples lying in the training set [46]. We have not carried out an investigation whether even a lower percentage of training data could have learnt the pattern or not, since a 50% split worked well in our explorations. The 50% split is a balanced treatment of the data partitioning for training/testing but has been carried out within the random 1000 Monte Carlo draws from the whole dataset. Using such equal amounts of training and testing data also shows a more rigorous testing where half of the whole dataset in a single Monte Carlo draw is sufficient to reliably train the classifiers and yield consistent performance metrics.

### 2.3 Outlier detection and removal

**Fig 2:**
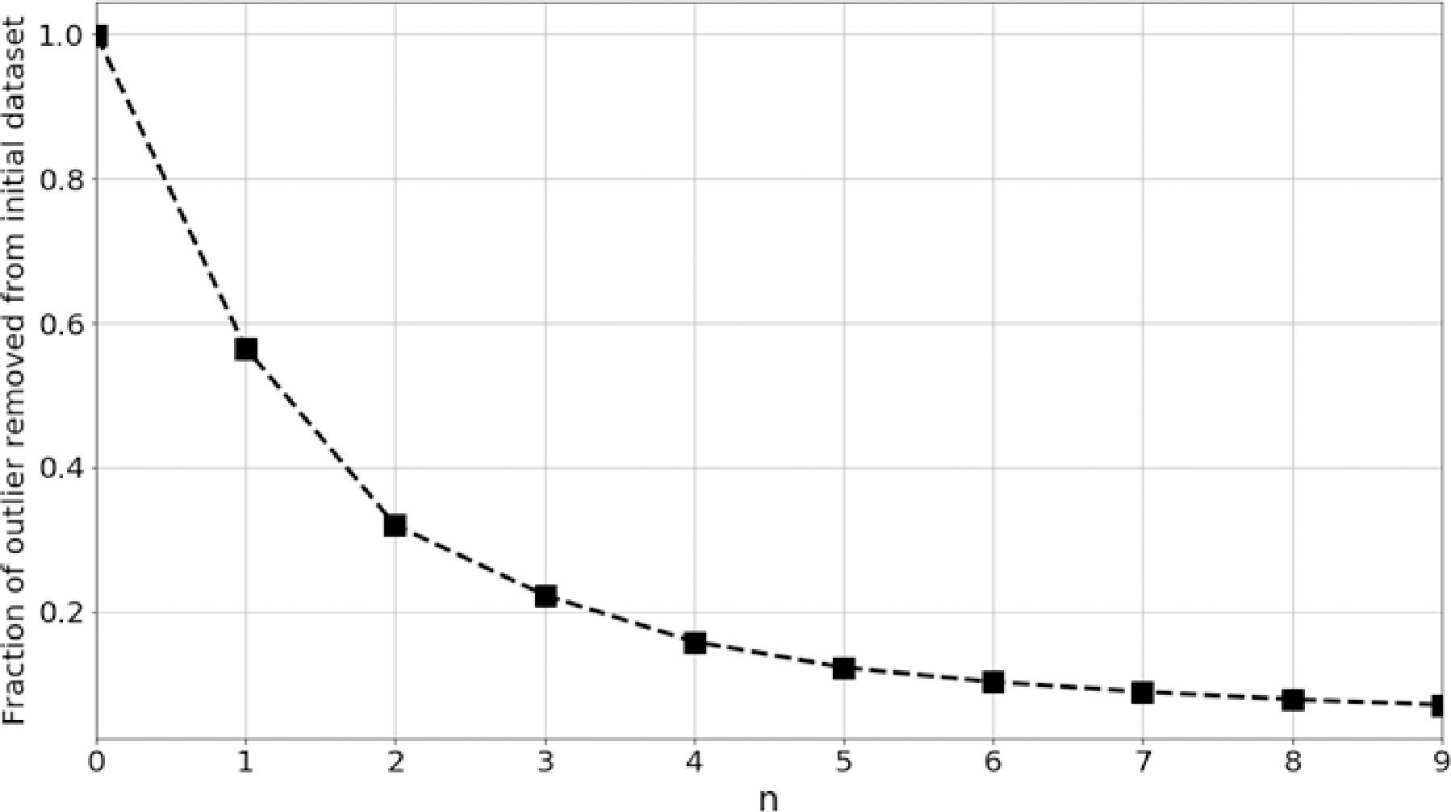
Outlier detection and removal effects on the percentage of the data retained. Fraction of outliers *n* = 6 has been selected to retain data without significant outliers.

We have detected outliers in the dataset by using the interquartile range (IQR). The IQR is defined as the first quartile subtracted from the third quartile. We have removed the outlier following the condition (*Q*1 –*n* × IQR) < data < (*Q*_1_ + *n* × IQR), where *n* represents an integer. In Fig 2, we have shown how the number of samples in the data is reduced as we vary *n*. We observe that when *n* = 6, almost 10% of the data is reduced. Therefore, we choose to keep the data between (*Q*1−6 × IQR) < data < (*Q*_1_ + 6 × IQR).

It is a standard practice to consider 1.5×IQR for detecting outliers only in perfect univariate Gaussian distributions. Since we do not know the distribution of the features beforehand, it is important to vary the multiplication factor of the IQR. We apply a uniform criterion on the samples, and it is not important here to keep track of the percentage of the removed datapoints in each class. This is because we are using a Monte Carlo strategy to make the classification a balanced one. The number *n* here just shows how much noisy samples, can we allow to be included in the classification process.

Outliers can bias the classifiers, so it is important to reject the extreme valued samples. Outliers occurred occasionally during the data collection using the current threshold and they are not related to AP or VP. Here we are extracting features from longer episodes as compared to the typical length of AP/VP [51]. Previously we designed optimal IIR filters for removing drift on plant electrophysiological signals [5]. However, the extracted features still may have some extreme values due to sudden change in the stochastic high frequency part of the filtered signal which can be potential source of outlier samples and should be removed during training the classifiers.

### 2.4 Feature correlation analysis, dimensionality reduction using principal component analysis

**Fig 3:**
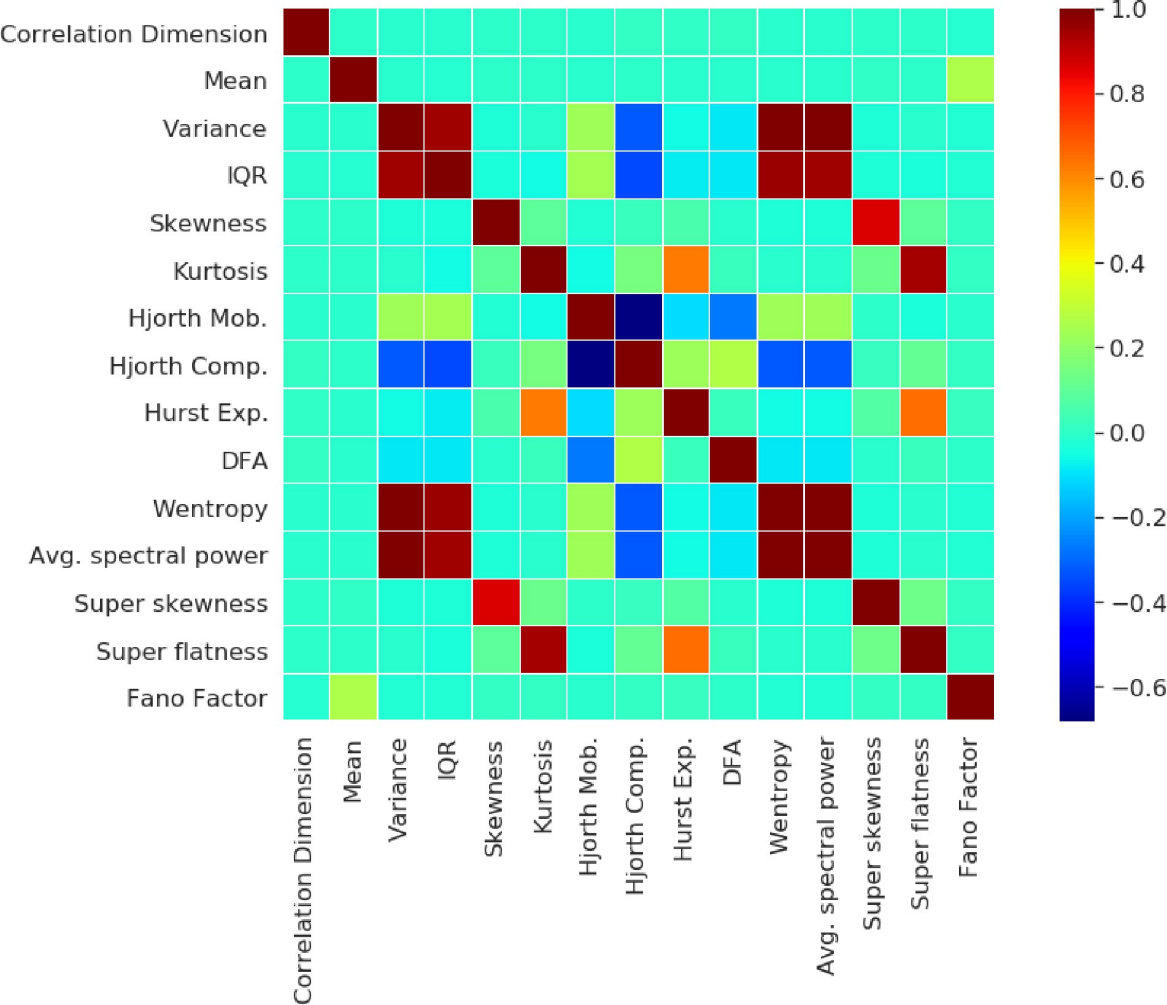
Heatmap of correlations amongst the 15 features after outlier detection. This shows the strong positive and negative correlations amongst the statistical features.

The correlation matrix between the 15 extracted features shows that many features have some degree of redundant information (expressed from the reddish/brownish hue for positive and bluish hue for negative correlations) as observed in Fig 3. Therefore, it becomes necessary to look at the correlation matrix of the features, as often we may introduce redundant information to the classifiers which can be removed or reduced using dimensionality reduction methods like PCA. This redundant information adds to the computation without adding value to the classification performance, and hence are removed.

**Fig 4:**
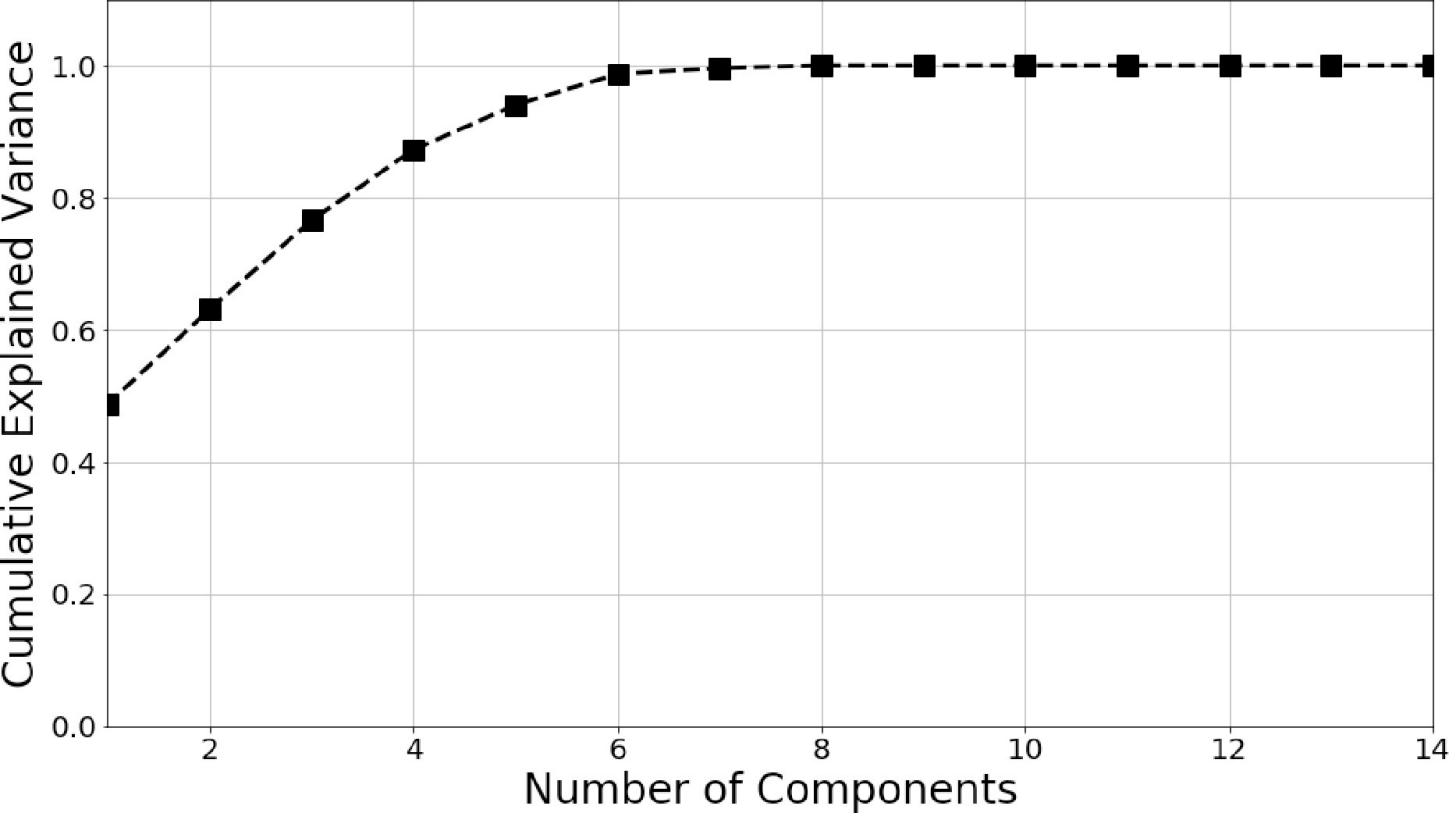
Scree plot showing the cumulative explained variance in PCA to select the number of PCs for the multiclass classification.

We also performed a PCA for dimensionality reduction in order to compare the classification results between the original feature space and the reduced PCA basis space. Fig 4 shows how the cumulative explained variance changes with the number of principal components (PCs). The percentage of variance explained is calculated as the sum of the first few strongest eigenvalues of the feature covariance matrix normalized by the sum of all eigenvalues of the covariance matrix and known as the scree plot shown in Fig 4. We observe that only 7 PCs represent > 90% cumulative explained variance. Therefore, we have chosen only the strongest 7 PCs for comparison with the unreduced high dimensional feature space.

Here, we have not carried out a feature selection/elimination technique, rather the use of PCA is to extract the useful information from all the 15 features and embed them into the 7 orthogonal PCs and removing the correlation between the original features. Each PC are some linear combinations of all the features and are orthogonal to each other. Selecting the number of PCs is done using the scree plot in Fig 4 when an acceptable percentage of variance explained is reached using the strongest PCs.

### 2.5 Handling unbalanced dataset using Monte Carlo under-sampling

We observe from Table 2, that we have a highly unbalanced multiclass dataset for classification. There are several methods to handle these kinds of unbalanced datasets as discussed in [46]^,^ [52–55]. As we observe that the data available has three classes with 628 (NaCl), 1488 (H_2_SO_4_) and 35718 (Ozone) datapoints respectively, we have taken a subset of the data with 628 points from each class using Monte Carlo under-sampling. Therefore, in each subset we have the whole dataset from NaCl and 628 points were chosen randomly from the other two classes. Thus, we prepare a balanced data set with 628 × 3 = 1884 datapoints and we performed this randomization and data shuffling 1000 times. Thereafter, we divide this subsample dataset of 1884 datapoints equally into training and testing sets. The performances of the models are computed by looking at the average, over 1000 ensembles of the confusion matrices, implementing different classification algorithms on these under-sampled datasets.

### 2.6 Hyperparameter settings for the classification algorithms

We have implemented 10 different classification models (from 8 different classification algorithm families) to compare and benchmark their performances in this plant stimuli classification problem. Table 3 lists the hyperparameters which have been varied as well as other parameters which are kept at their default values. The parameters which are not mentioned in Table 3 were kept at their default values. For each family of classification algorithms, one can carry out detailed hyperparameter optimization [37]. However, the focus of this paper is to compare the classification performance under original and reduced features under random under-sampling and with the same hyperparameters. A random search based hyperparameter tuning in each family of classifier will massively increase the computational burden and hence has been avoided here. However, the hyperparameters were tuned offline with a trial-and-error method to ensure the classification performance is acceptable and consistent in both the reduced seven-dimensional (7D) and original fifteen-dimensional (15D) feature sets. Optimal hyperparameter tuning using an optimization approach has been left as the scope of future work due to its high computational demand. The next section shows the average confusion matrices out of the 1000 ensembles in the Monte Carlo under-sampling classification task.

**Table 3:** Hyperparameters used in Scikit-learn package in Python [56], including both the default and customized values yielding robust classification on both the 15D and 7D feature space.

Classification Algorithm	Chosen Hyperparameters
AdaBoost	base_estimator = None specified—(DecisionTreeClassifier(max_depth = 5)) (default), n_estimators = 600, learning_rate = 50, algorithm = SAMME.R (default), random_state = None (default)
Decision tree	criterion = gini (default), splitter = best (default), max_depth = None (default), min_samples_split = 2 (default), min_samples_leaf = 1 (default)
Gaussian Naive Bayes (GNB)	Priors = None specified (adjusted according to the data), var_smoothing = 1e-9 (default)
*k*-nearest neighbour (*k*-NN)	n_neighbors (*k*) = 5 (default), weights = uniform (default), algorithm = auto (default), leaf_size = 30 (default), *p* = Power parameter for the Minkowski metric = 2 (default: Euclidean distance)
Multilayer perceptron classifier (MLPC)	hidden_layer_sizes = (100) (default), activation = relu (default), solver = adam (default), alpha = 2, batch_size = auto (default)
Quadratic discriminant analysis (QDA)	Priors = None, reg_param = 0, store_covariance = False, store_covariances = None, tol = 0.0001
Random Forest (entropy)	n_estimators = 100 (default), criterion = entropy, max_depth = 5, min_samples_split = 2 (default), class_weight = balanced
Random Forest (gini)	n_estimators = 100, criterion = gini, max_depth = 5, min_samples_split = 2 (default), class_weight = None (default)
Support vector machine (linear)	*C* = 1 (default), kernel = linear, degree = 3 (default), gamma = 1.5, decision_function_shape = ovr (default)
Support vector machine (RBF)	*C* = 1.0 (default), kernel = rbf, degree = 3 (default), gamma = 1.5, decision_function_shape = ovr (default)

### 2.7 Classification performance measures for unbalanced multiclass dataset

There have been many studies on classification performance metrics based benchmarking for both binary and multiclass problems with almost equal number of datapoints in each class to highly skewed unbalanced classification problems [57]. We start with simple classification measures like average accuracy derived from the stacked confusion matrices in the under-sampling scheme to more advanced metrics like balanced accuracy, F_1_ score and Matthews correlation coefficient and also the relationships between these metrics, as described below.

Let us-consider the average confusion matrix for the unbalanced dataset over 1000 Monte Carlo under-sampling of the majority classes is represented by:

$$\bar{C}=[\begin{array}{ccc} {\bar{c}}_{11} & {\bar{c}}_{12} & {\bar{c}}_{13}\\ {\bar{c}}_{21} & {\bar{c}}_{22} & {\bar{c}}_{23}\\ {\bar{c}}_{31} & {\bar{c}}_{32} & {\bar{c}}_{33} \end{array}]=\frac{1}{n}\sum_{i=1}^{n}\left[\begin{array}{ccc} c_{{}_{11}}^{i} & c_{{}_{12}}^{i} & c_{{}_{13}}^{i}\\ c_{{}_{21}}^{i} & c_{{}_{22}}^{i} & c_{{}_{23}}^{i}\\ c_{{}_{31}}^{i} & c_{{}_{32}}^{i} & c_{{}_{33}}^{i} \end{array}\right],n=1000.$$ (1)

The classification accuracy for the three-class problem is calculated as:

$${\mathrm{Acc}}_{i}=\left(\frac{c_{11}^{i}}{\sum_{j=1}^{3}c_{1j}^{i}}+\frac{c_{22}^{i}}{\sum_{j=1}^{3}c_{2j}^{i}}+\frac{c_{33}^{i}}{\sum_{j=1}^{3}c_{3j}^{i}}\right)/3=\frac{1}{3}\sum_{k=1}^{3}\left(\frac{c_{kk}^{i}}{\sum_{j=1}^{3}c_{kj}^{i}}\right),i=1,\cdots ,1000.$$ (2)

The above metric is not always robust against high class imbalance and non-Gaussian heterogeneity in the dataset which can be better captured using the three performance measures viz. balanced accuracy, F_1_ score and Matthews correlation coefficient (MCC). It has been suggested in [58] that the MCC being the best classification performance evaluation metric for binary classification problems, especially for unbalanced datasets. The balanced accuracy is defined in terms of the class-specific sensitivity (Sen) and specificity (Spe) measures as:

$$\begin{array}{l} {\mathrm{BalAcc}}_{i}=\frac{1}{n_{class}}(\sum_{i=1}^{n_{class}}\frac{{\mathrm{Sen}}_{i}+{\mathrm{Spe}}_{i}}{2})\\ =\frac{1}{2n_{class}}\left(\sum_{i=1}^{n_{class}}\left[\left(\frac{\sum_{j=1}^{n_{class}}{\mathrm{TP}}_{j}}{\sum_{j=1}^{n_{class}}{\mathrm{TP}}_{j}+\sum_{j=1}^{n_{class}}{\mathrm{FN}}_{j}})+(\frac{\sum_{j=1}^{n_{class}}{\mathrm{TN}}_{j}}{\sum_{j=1}^{n_{class}}{\mathrm{TN}}_{j}+\sum_{j=1}^{n_{class}}{\mathrm{FP}}_{j}}\right)\right]\right),i=1,\cdots ,1000. \end{array}$$ (3)

Here, $\{{\mathrm{TP}}_{j},{\mathrm{TN}}_{j},{\mathrm{FP}}_{j},{\mathrm{FN}}_{j}\},j=1,\ldots ,n_{class}$ represent the class-specific true positive, true negative, false positive and false-negative detections. The balanced accuracy in (3) considers the misclassifications by using the sensitivity and specificity metrics for each class, thereby not only relying on the diagonal of the confusion matrix as in the standard multi-class accuracy measure in (2). Hence it serves as a more accurate and robust measure of a classifier’s performance.

The Matthews correlation coefficient for multi-class classification problem is defined in terms of the confusion matrix *C* for multiple classes (*n*_*class*_) as:

$$\mathrm{MCC}=\frac{c\times s-\sum_{k}^{n_{class}}p_{k}\times t_{k}}{\sqrt{\left(s^{2}-\sum_{k}^{n_{class}}p_{k}^{2})\times (s^{2}-\sum_{k}^{n_{class}}t_{k}^{2}\right)}}.$$ (4)

Here in (4), the variables are defined as:

- $t_{k}=\sum_{i}^{n_{class}}C_{ik}$ being the number of times class *k* truly occurred,
- $p_{k}=\sum_{i}^{n_{class}}C_{ki}$ being the number of times class *k* was predicted,
- $c=\sum_{k}^{n_{class}}C_{kk}$ being the total number of samples correctly predicted,
- $s=\sum_{i}^{n_{class}}\sum_{j}^{n_{class}}C_{ij}$ being the total number of samples.

The F_1_ score represents the weighted harmonic mean of the class-specific precision (Pre) and recall (Rec) for the multi-class problem and is defined as:

$$F_{\beta }=(1+\beta^{2})\frac{\mathrm{Pre}\times \mathrm{Rec}}{\beta^{2}\mathrm{Pre}\times \mathrm{Rec}},$$ (5)

where, ${\mathrm{Pre}}_{i}={\mathrm{TP}}_{i}/\left({\mathrm{TP}}_{i}+{\mathrm{FP}}_{i}\right),{\mathrm{Rec}}_{i}={\mathrm{TP}}_{i}/({\mathrm{TP}}_{i}+{\mathrm{FN}}_{i}),i=1,\cdots ,n_{class}$. Putting *β* = 1 in the generic expression (5) yield the overall F_1_-score, representing that both precision and recall for each class are equally important. These performance metrics capture various aspects of the classification results and are complementary to each other. Other research on multiclass highly unbalanced data [59] shows the use of hybrid performance measures using these various metrics to be advantageous [60].

## 3. Results and discussions

**Fig 5:**
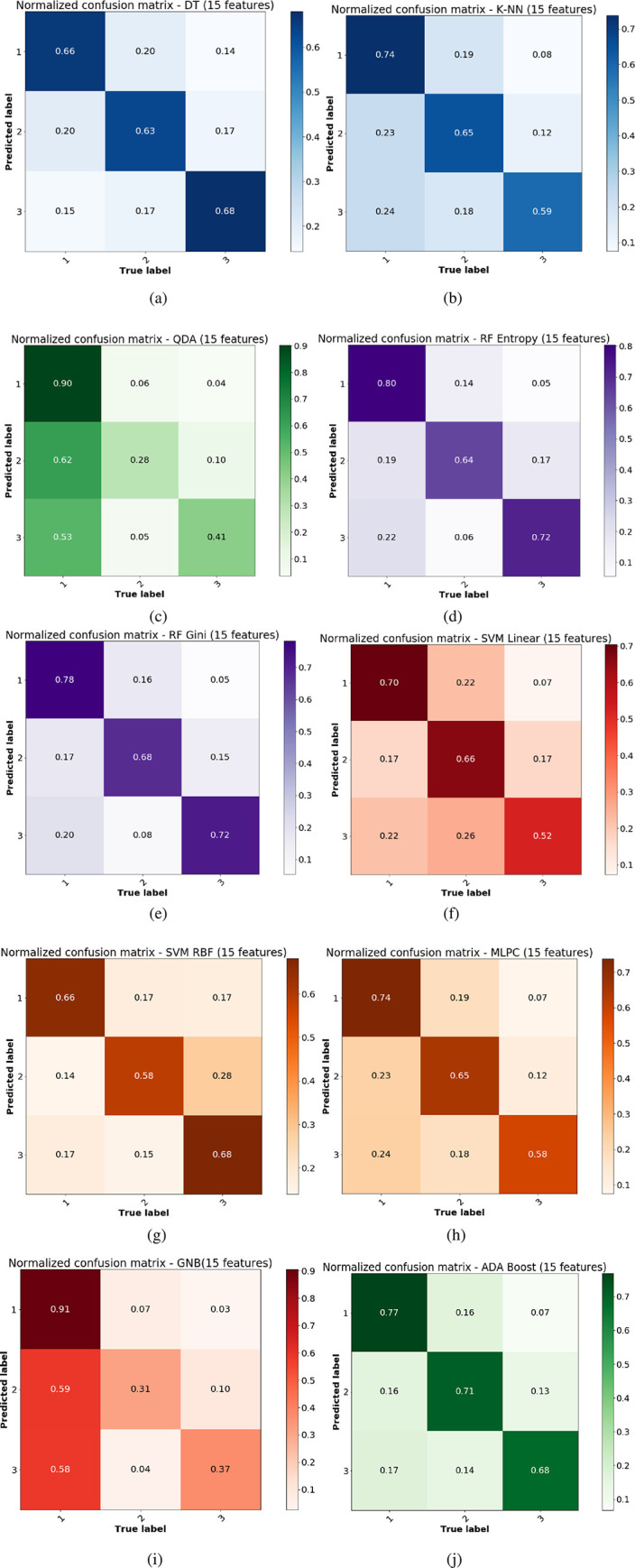
Average normalized confusion matrices over 1000 Monte Carlo draws from the majority class using the original feature and various classifiers in the original 15D feature space. Mean confusion matrices are calculated from the held out 50% test data under each draw of the 1000 Monte Carlo under-sampling with different classifiers: (a) decision tree, (b) *k*-NN, (c) QDA, (d) Random forest (entropy) (e) Random forest (gini), (f) SVM (linear kernel) (g) SVM (RBF kernel), (h) MLP classifier, (i) Gaussian Naïve Bayes, (j) Adaptive boosting algorithm.

**Fig 6:**
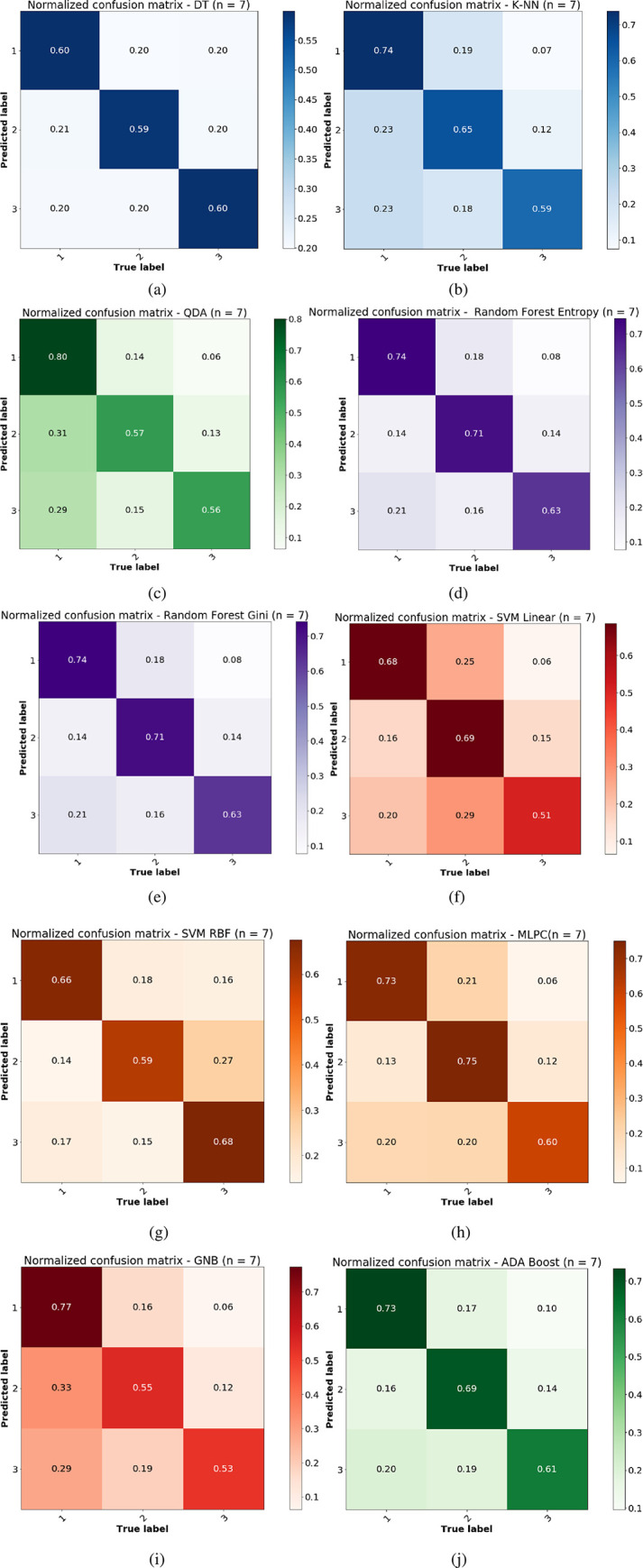
Average normalized confusion matrices over 1000 Monte Carlo draws from the majority class using the original feature and various classifiers in the reduced 7D feature space using PCA. Mean confusion matrices are calculated form the held out 50% test data under each draw of the 1000 Monte Carlo under-sampling with different classifiers: (a) decision tree, (b) *k*-NN, (c) QDA, (d) Random forest (entropy) (e) Random forest (gini), (f) SVM (linear kernel) (g) SVM (RBF kernel), (h) MLP classifier, (i) Gaussian Naïve Bayes, (j) Adaptive boosting algorithm.

**Fig 7:**
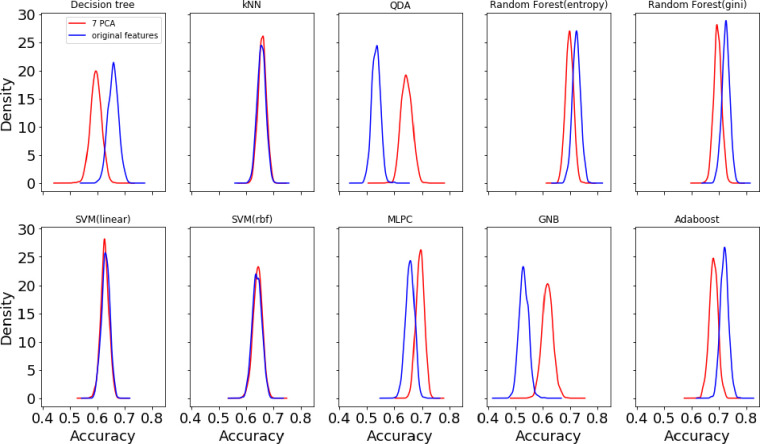
Comparison of the distributions of the *classification accuracies* derived from the ensemble of confusion matrices over 1000 Monte Carlo under-sampling runs of the majority class in the original 15D and reduced 7D feature space.

The Monte Carlo under-sampling was carried out 1000 times to create ensemble subsets of the whole dataset from three classes with equal size to make sure that the classification problem is turned into a balanced one before feeding into a chosen family of classifier. Due to the inherent randomness in the classification process, the confusion matrices are expected to vary by small amounts. Therefore, we here report the average confusion matrices in the original 15D feature space and the reduced 7D version using PCA in Figs 5 and 6 respectively. We also report the average classification performance which is the trace or sum of diagonals of individual confusion matrices normalized by the class number in Fig 7. The distribution of accuracies can be compared between the original 15D vs reduced 7D version of the classification problem in Fig 7, where the variances between the ensembles are found to be quite small, showing the robustness of our data analysis pipeline. The histogram of the accuracies derived from the 1000 ensembles of the confusion matrices in Fig 7 show that the random forest (gini) and the Adaboost algorithms both yield high classification accuracy. Here, all computational experiments for machine learning benchmarking were run on Python language on a 64-bit Linux PC with 4 parallel cores, 7.7 GB memory and Intel i5-6200U, 2.3 GHz processor. The feature extractions were carried out in Matlab as detailed in [3, 4, 6] using the MATS toolkit [29].

### 3.1 Univariate distributions of the statistical features and class separability

Since all the 15 features were used in training different family of classifiers, feature importance analysis or feature selection was not relevant here. However, for better interpretability of the classification results based on the individual species used and the chemical stimulus, we here provide univariate distributions of all the 15 features for each case similar to the analysis reported in [4] and based on two different plant species. We now show histograms of all the features for different species and stimulus. The univariate distributions of the discriminatory features clearly show that the separability between the classes (stimulus) and also the species. Although some of them may have some degree of overlap which shows the need for complex classifiers and the use of higher number of features.

**Fig 8:**
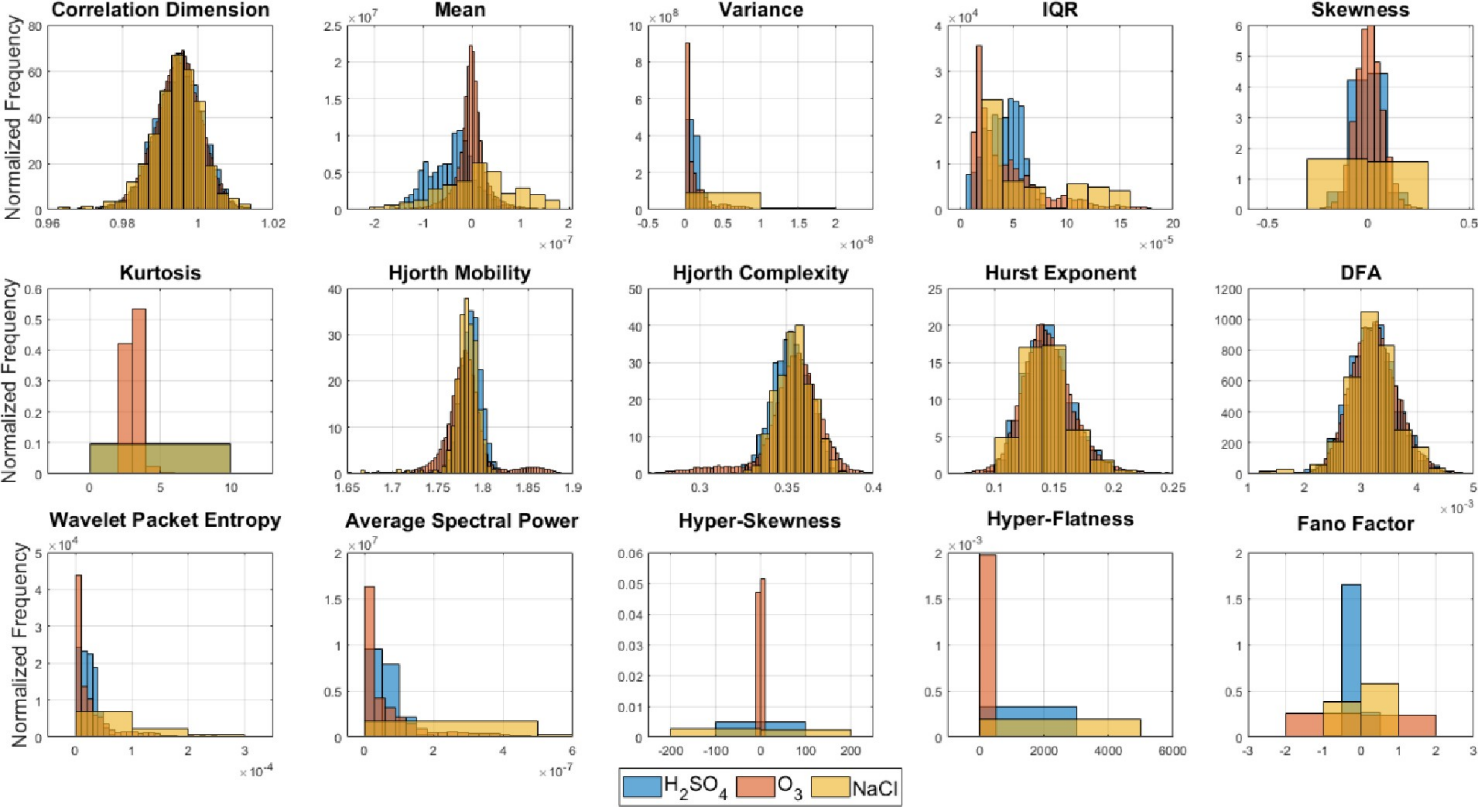
Normalized histograms of the three different chemical stimuli (sulfuric acid, ozone, and salt) for all 15 statistical features used.

**Fig 9:**
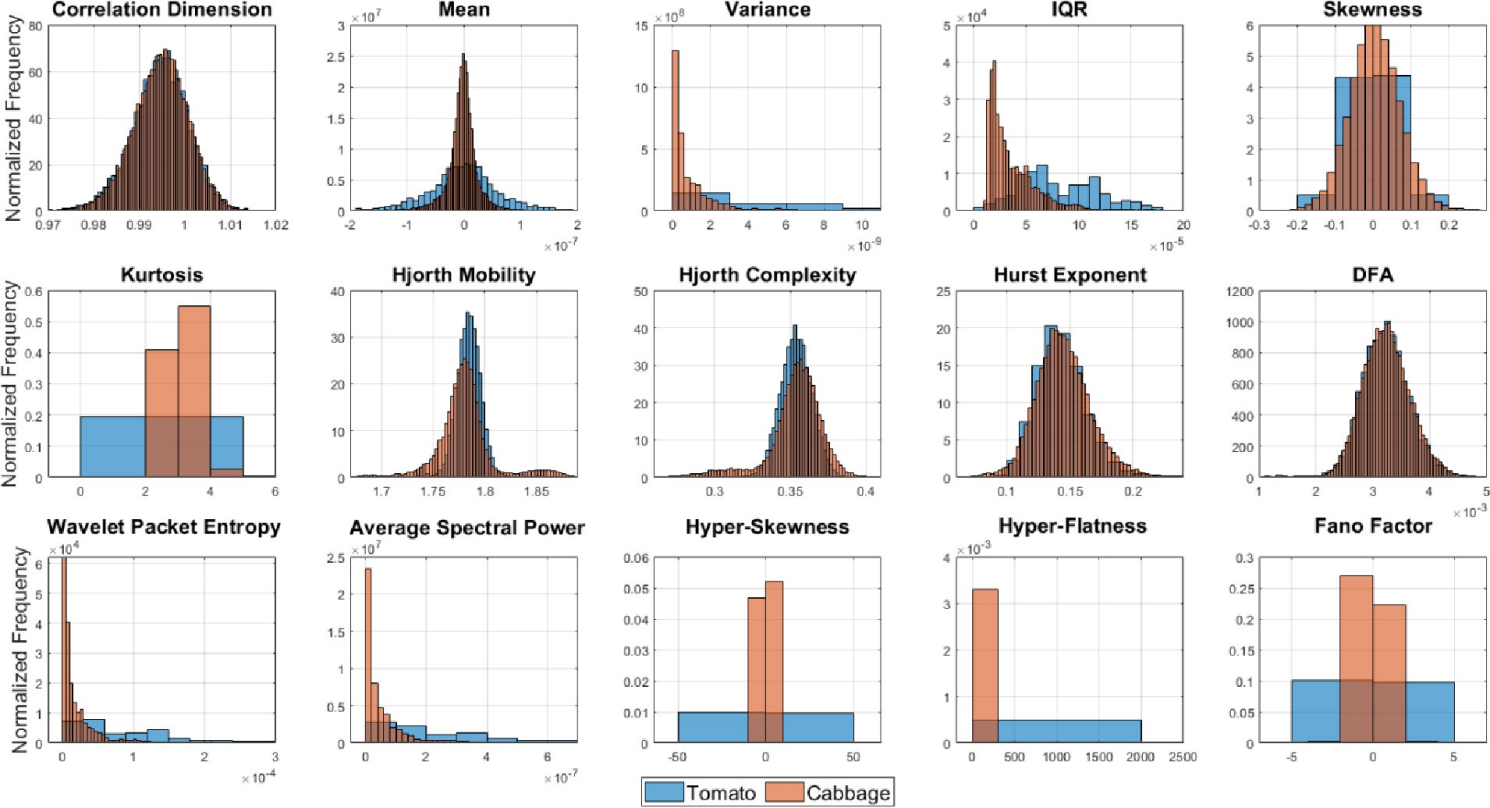
Normalized histograms of the two different plant species (tomato and cabbage) for all 15 statistical features used.

However, it is evident that there is large degree of overlap in the histograms of the three classes (or chemical stimuli) in Fig 8 which indicate that using simple statistical features it is not possible to separate them easily. Similar histograms for the two plant species—tomato and cabbage in Fig 9 also shows that there is large overlap using most features which indicate that the dataset do not introduce any bias due to a specific species. Fig 9 also shows that some of the feature like variance, IQR, kurtosis, hyperskewness, hyper-flatness and Fano factor yield wider spread of the distribution for the tomato species as compared to the cabbage. This justifies that the inclusion of more plant species which renders a robust classification strategy instead of using only one species.

**Fig 10:**
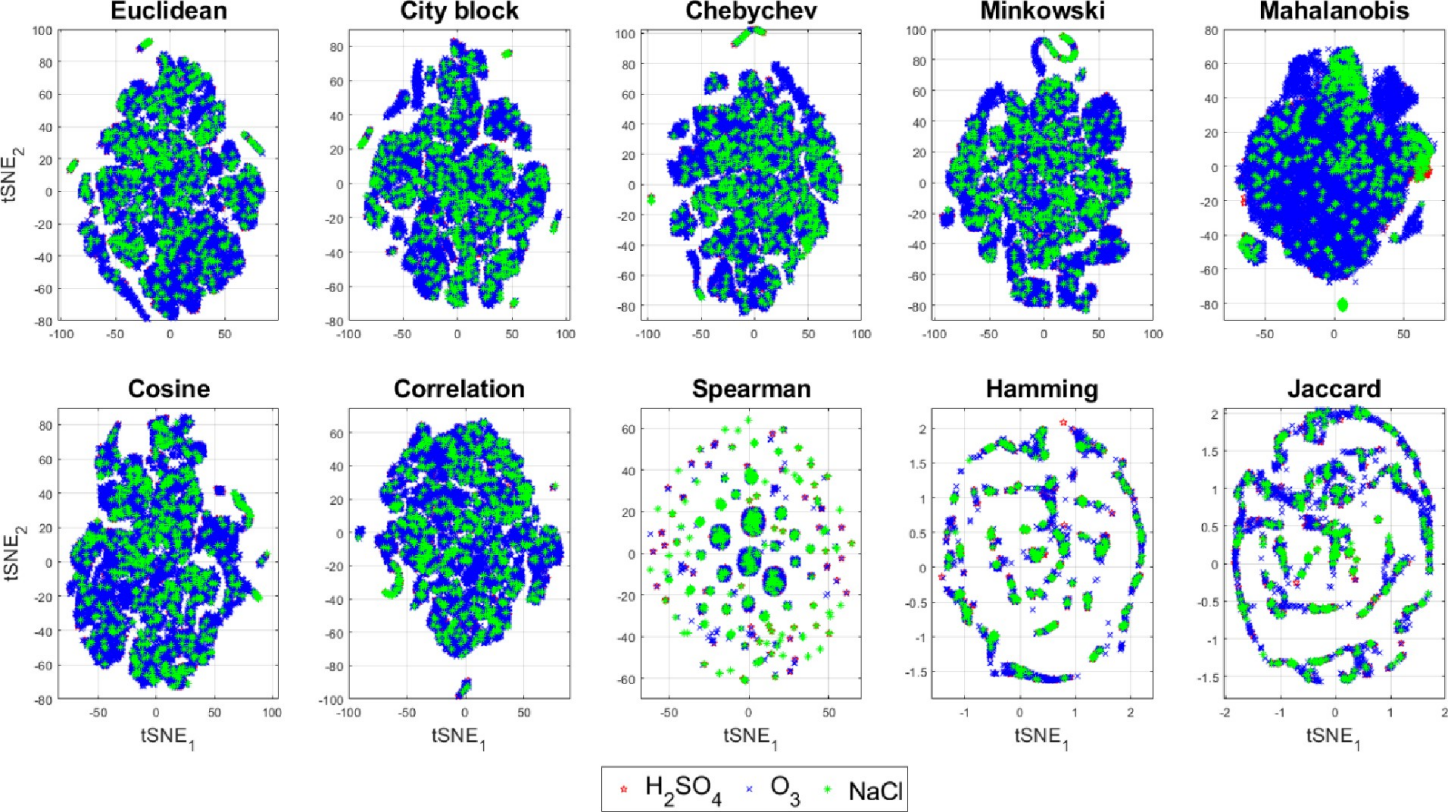
Reduced dimensional t-SNE plot of the three different chemical stimuli (sulfuric acid, ozone, and salt).

**Fig 11:**
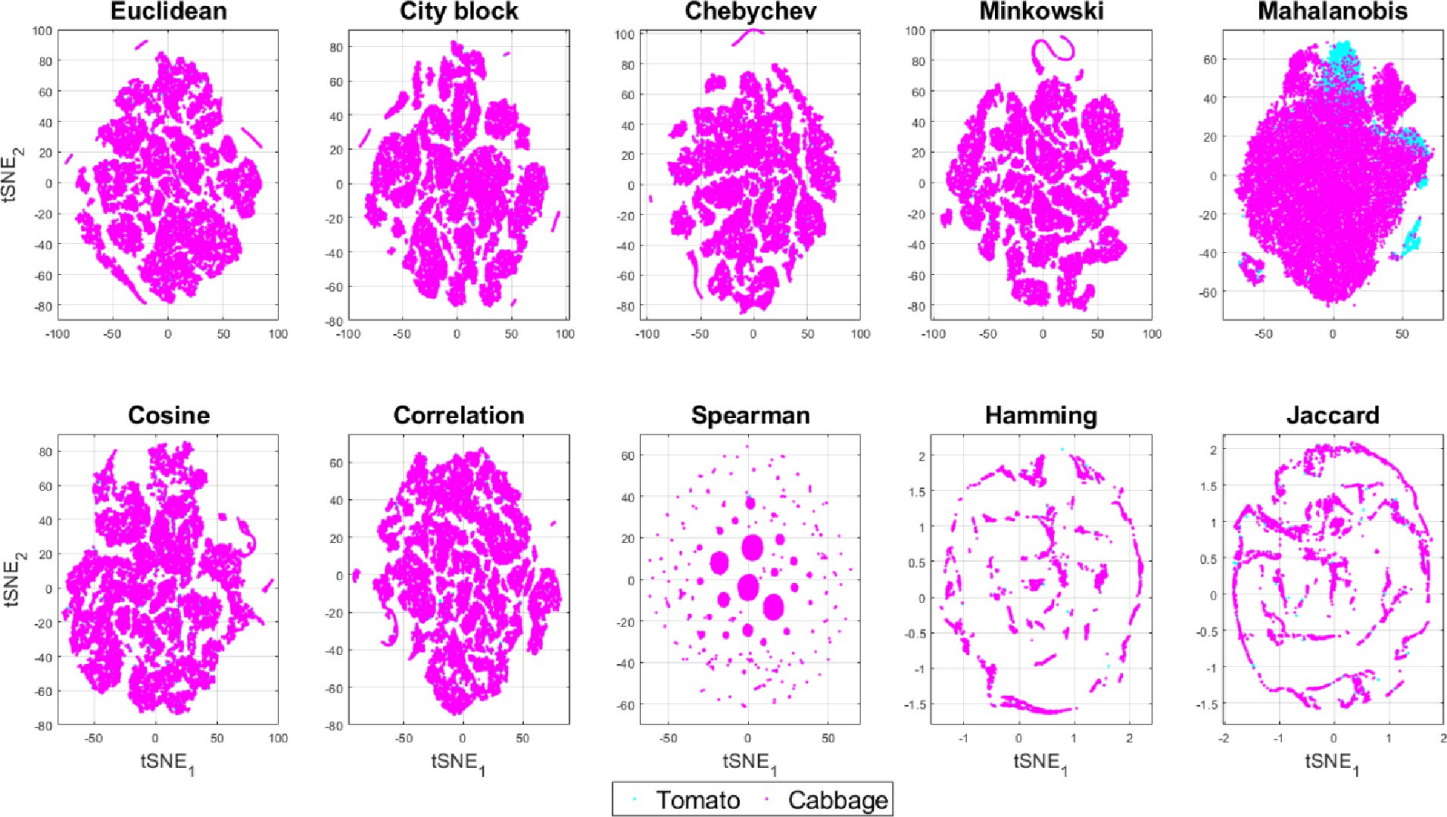
Reduced dimensional t-SNE plot of the two plant species tomato and cabbage.

Since using simple features and their simpler combinations are not able to classify the stimuli so easily, we next looked at the lower dimensional projection of all the 15 features explored using a 2D *t*-distributed stochastic neighbor embedding (*t*-SNE) plot. In the 2D *t*-SNE plots, we use 10 different distance metrics–Euclidean, city-block, Chebyshev, Minkowski, Mahalanobis, cosine, correlation, Spearman, Hamming and Jaccard for discriminating the three chemical stimuli in Fig 10 and the two plant species in Fig 11. It is evident from these reduced dimensional *t*-SNE plots that only the Mahalanobis distance shows less overlap and slightly better separation of the classes and species as compared to the rest 9 distance metrics as also found in [61, 62]. This may be due to the fact that the multivariate dispersion of the features i.e. covariance are different for the chemical stimuli and plant species although their central tendencies (e.g. median or mode) may be similar which can be better captured in the Mahalanobis distance as opposed to other distance metrics.

Since we aim to build complex classifiers in a multidimensional feature space, it may not always be possible to interpret the classification results in terms of individual features’ contribution and associate them with the class labels as evident from the histograms. However, we next report a thorough exploration of classification in the original 15D vs. 7D reduced space using the strongest principal components. Biological and chemical interpretation of the feature importance is beyond the scope of this work. However, for better interpretation we show histogram plots for different features used in the classification for different species and chemical stimuli which is comparable with the previous results reported in Chatterjee *et al*. [4]. Although many of these histograms have overlapping regions, still they show the distinct areas as a signature of a particular stimulus-species-feature based class separability.

### 3.2 Classification in the original high dimensional feature space

Using the 15 higher order statistical features extracted from the plant signals, the classification performances in the balanced under-sampled setting for all the three classes are shown in Fig 5, using 10 different classifier settings. Most of the classifiers, except QDA and GNB, are able to yield reasonably good classification accuracy as evident from the lighter shades in the non-diagonal elements of their respective confusion matrices. Due to the Monte Carlo under-sampling approach for tackling the unbalanced dataset, the confusion matrices here represent the average classification performance for the 1000 ensemble or subset of data drawn randomly from the majority classes. The classification results are reported in a normalized scale of (0–1) instead of reporting the number of correct and incorrect classification instances for better interpretability.

### 3.3 Classification in the reduced dimension using PCA

A similar classification benchmarking is done using the strongest 7 PCs and the same set of classifiers as the original high dimensional feature space. This exploration would help us to understand whether the dimensionality reduction using PCA helps in improving the classification accuracy by compactly representing the data in lower dimensional space vs. the amount of information lost in the PCA. Similar to the original 15D feature space, the GNB and QDA are found to perform the worst having higher mis-classification rate as compared to the other classifiers, in the 7D feature space. However, both QDA and GNB performed better in the reduced feature space as compared to the original higher dimensional feature space. In the next sub-section, we report the mean and standard deviation of the ensemble of 1000 confusion matrices for different models and two different feature representations i.e. in the original 15D space and the reduced 7D space of the PCs.

### 3.4 Comparison of classification performance in the original and reduced feature space

Fig 7 shows the distribution of the classification performance from the ensemble of confusion matrices over the 1000 Monte Carlo draws of the majority class showing the average and dispersion of the achievable accuracies by each of the eight families of classifiers, indicating the consistency of the classification methods. The narrow standard deviation of the classification accuracies in Fig 7 shows good robustness properties of the classifiers and features with the best-found hyperparameter settings for each classifier family between the reduced PCA based (7D) and the original 15D feature space. The difference between the 7D vs. 15D are insignificant in the case of *k-*NN and SVM classifiers with linear and RBF kernels. The dimensionality reduction improves the performance as compared to the original feature space in the case of QDA, MLP and GNB classifiers. However, in other cases i.e. decision tree, random forest with gini/entropy and Adaboost classifiers, the original 15D feature space yields better discrimination performance. Overall, the best classification performance is achieved by the random forest (gini/entropy) followed by Adaboost classifier in the original 15D feature space.

**Fig 12:**
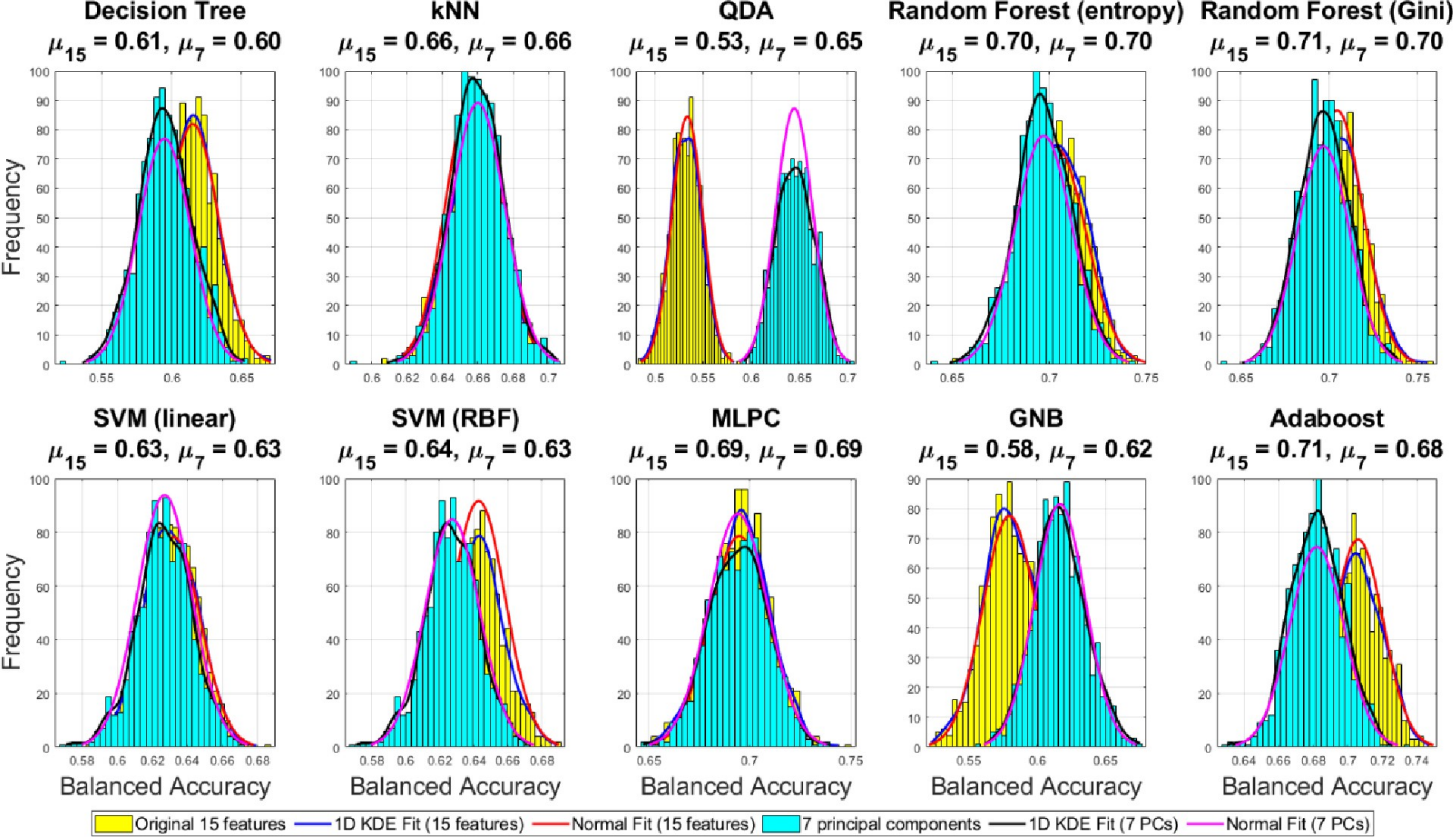
Comparison of the distributions of the balanced accuracies derived from the ensemble of confusion matrices over 1000 Monte Carlo under-sampling runs of the majority class in the original 15D and reduced 7D feature space with 1D KDE fit and normal fit to compare the mean difference.

**Fig 13:**
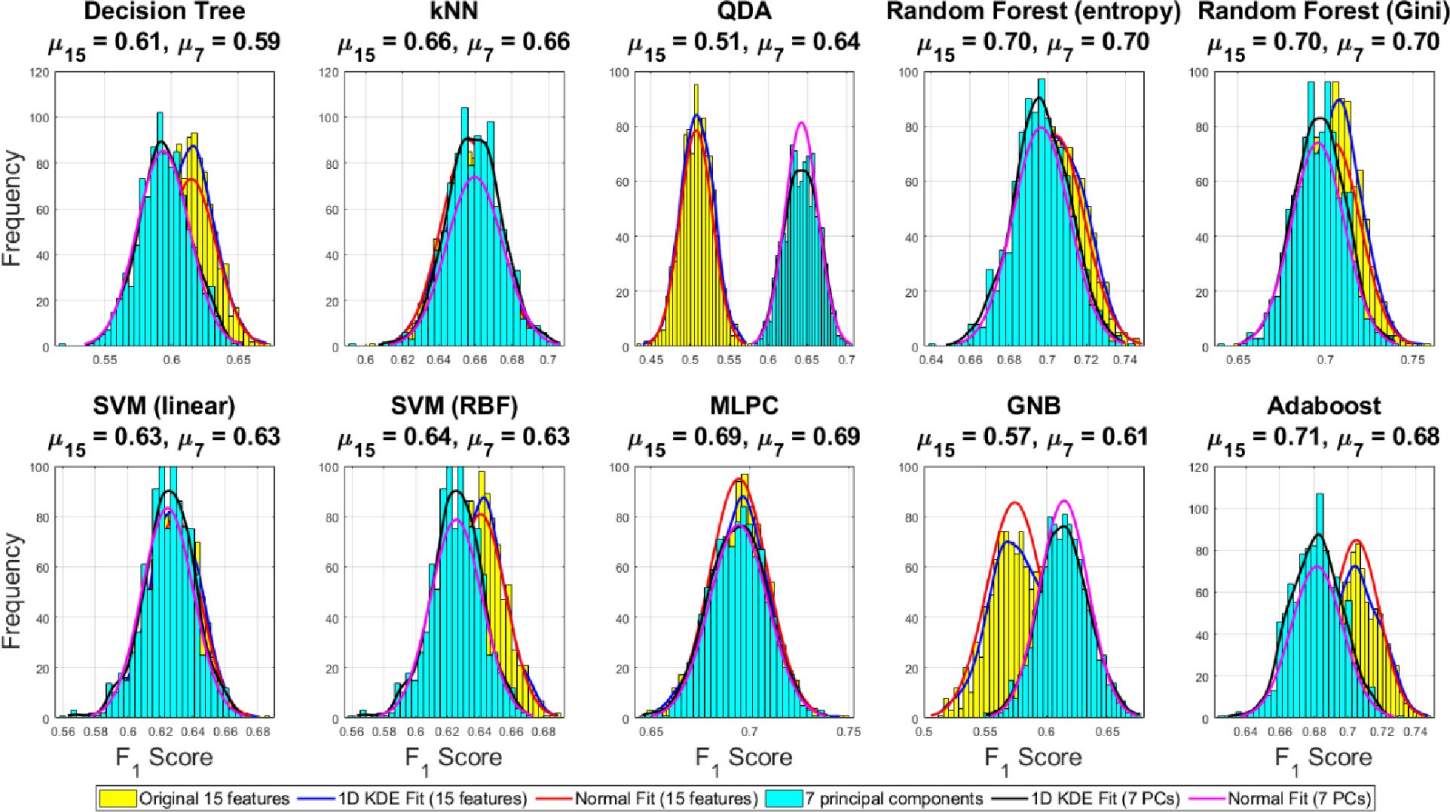
Comparison of the distributions of the F_1_ score derived from the ensemble of confusion matrices over 1000 Monte Carlo under-sampling runs of the majority class in the original 15D and reduced 7D feature space with 1D KDE fit and normal fit to compare the mean difference.

**Fig 14:**
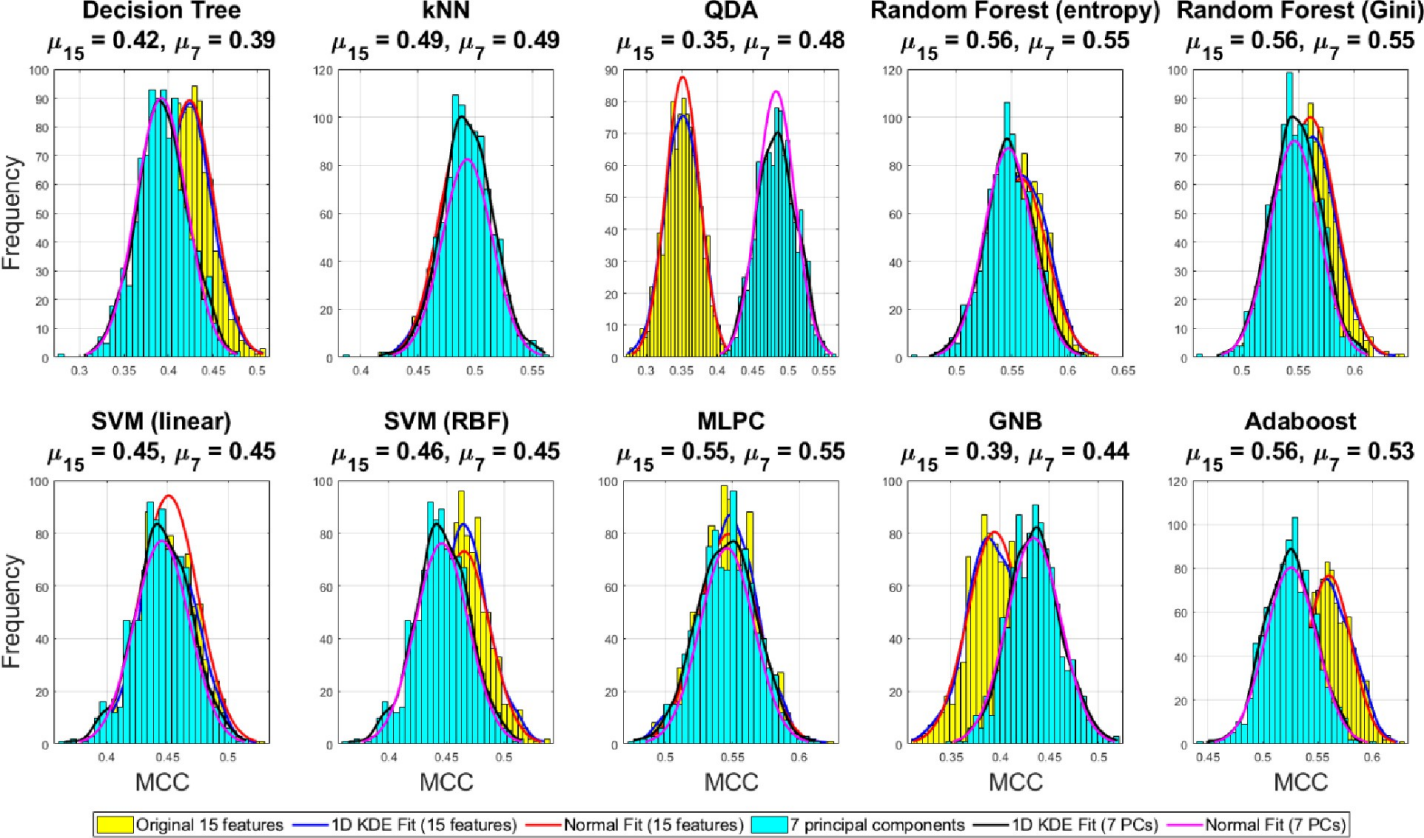
Comparison of the distributions of the Matthews correlation coefficient derived from the ensemble of confusion matrices over 1000 Monte Carlo under-sampling runs of the majority class in the original 15D and reduced 7D feature space with 1D KDE fit and normal fit to compare the mean difference.

However, as discussed before, the standard classification accuracy derived from the confusion matrices may not be the ideal performance measure for comparison in the case of highly unbalanced datasets and with more than two classes. Therefore, we have reported three other classification performance metrics widely applicable for unbalanced multi-class problems viz. F_1_ score, MCC, and balanced accuracy. We also report the 1D kernel density estimate (KDE) fit and equivalent normal fit of the histograms for the three performance-metrics using the 1000 ensemble runs under the Monte Carlo under-sampling framework. Mean of the fitted normal fit reported on the title of each subplot in Figs 12–14 allows clearer comparison of the achievable performance gain between the original 15D feature space vs 7D reduced PC space using all chosen family of classifiers.

Fig 12 shows that the random forest (gini) and Adaboost classifiers yielding the highest balanced accuracy using the 7D reduced features as compared to other classifiers and the original 15D features. However, the higher dimensional classification yields better results with QDA and GNB classifiers. However, slightly better balanced-accuracy is achieved for the decision tree and Adaboost classifiers. The best balanced-accuracy = 0.71 is achieved in the original 15D feature space using both the random forest (Gini) and Adaboost classifiers outperforming all other cases.

Histograms of the F_1_ score and MCC over 1000 Monte Carlo under-sampling-based classification have been shown in Figs 13 and 14 respectively. The best F_1_ score = 0.71 is achieved by the Adaboost classifier using the 15 original features which outperforms all the other cases. The conservative measure MCC = 0.56 is highest using Adaboost and both the random forest (gini and entropy) classifiers in the original 15D feature space as compared to the reduced 7 PCs. Therefore, combining all the three metrics relevant for the unbalanced dataset classification problems, we can conclude that the best performance has been achieved by the Adaboost classifier with the original 15D feature space where it outperforms all other classifiers in one or more performance metrics.

**Fig 15:**
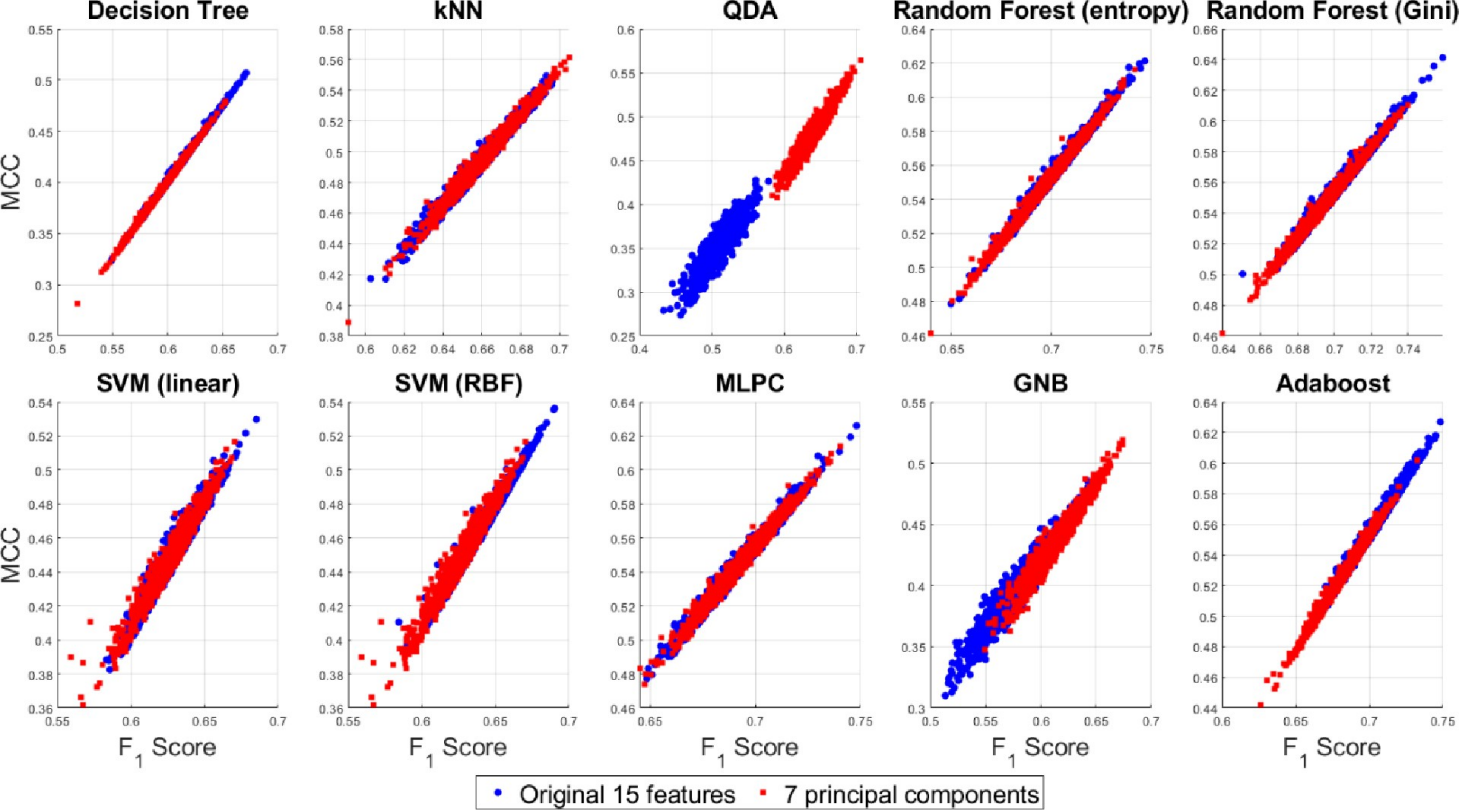
Scatterplots between the two performance measures–F_1_ score vs. MCC for the original 15D and 7D feature-based classification with different classifier families.

**Fig 16:**
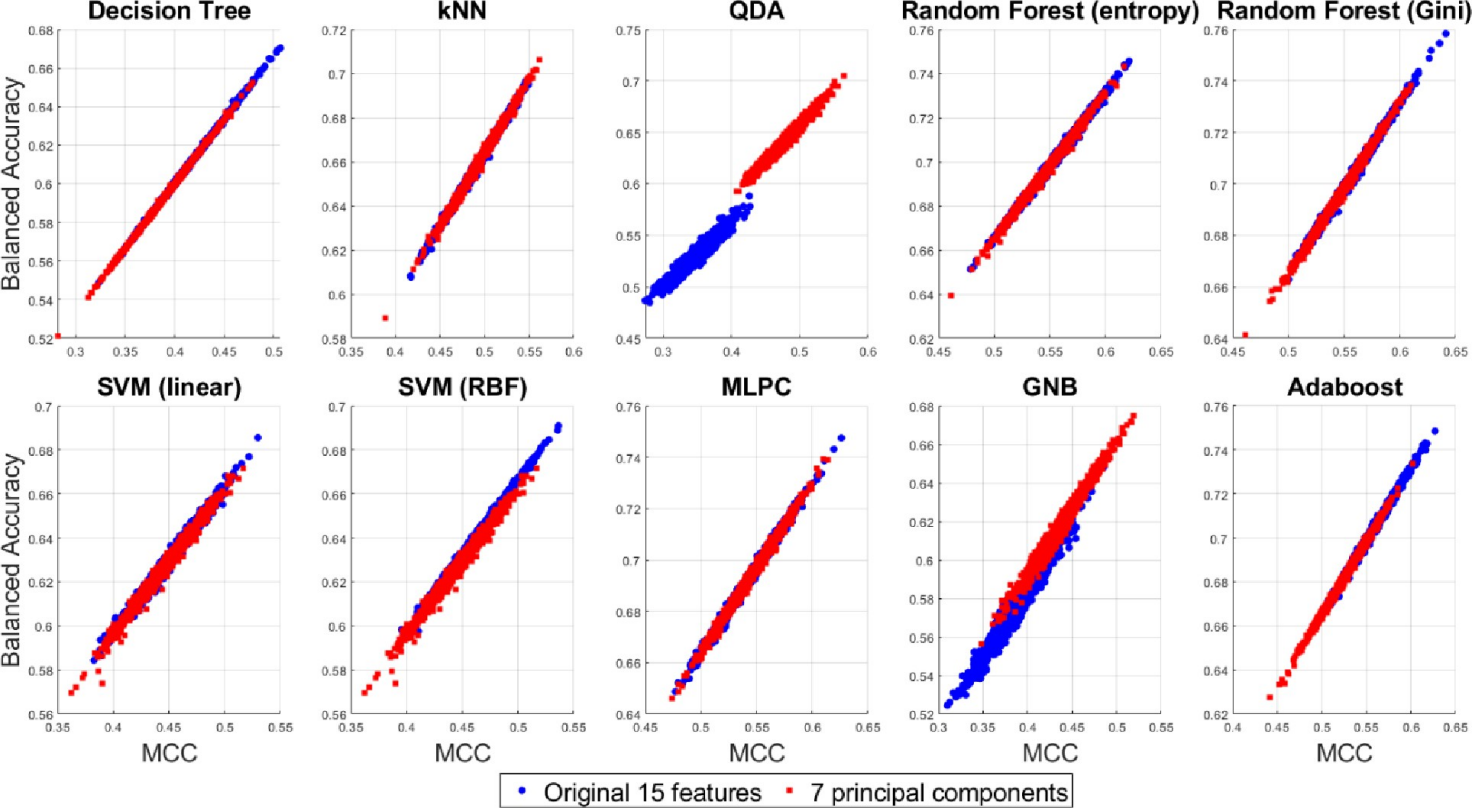
Scatterplots between the two performance measures–MCC vs. balanced accuracy for the original 15D and 7D feature-based classification with different classifier families.

**Fig 17:**
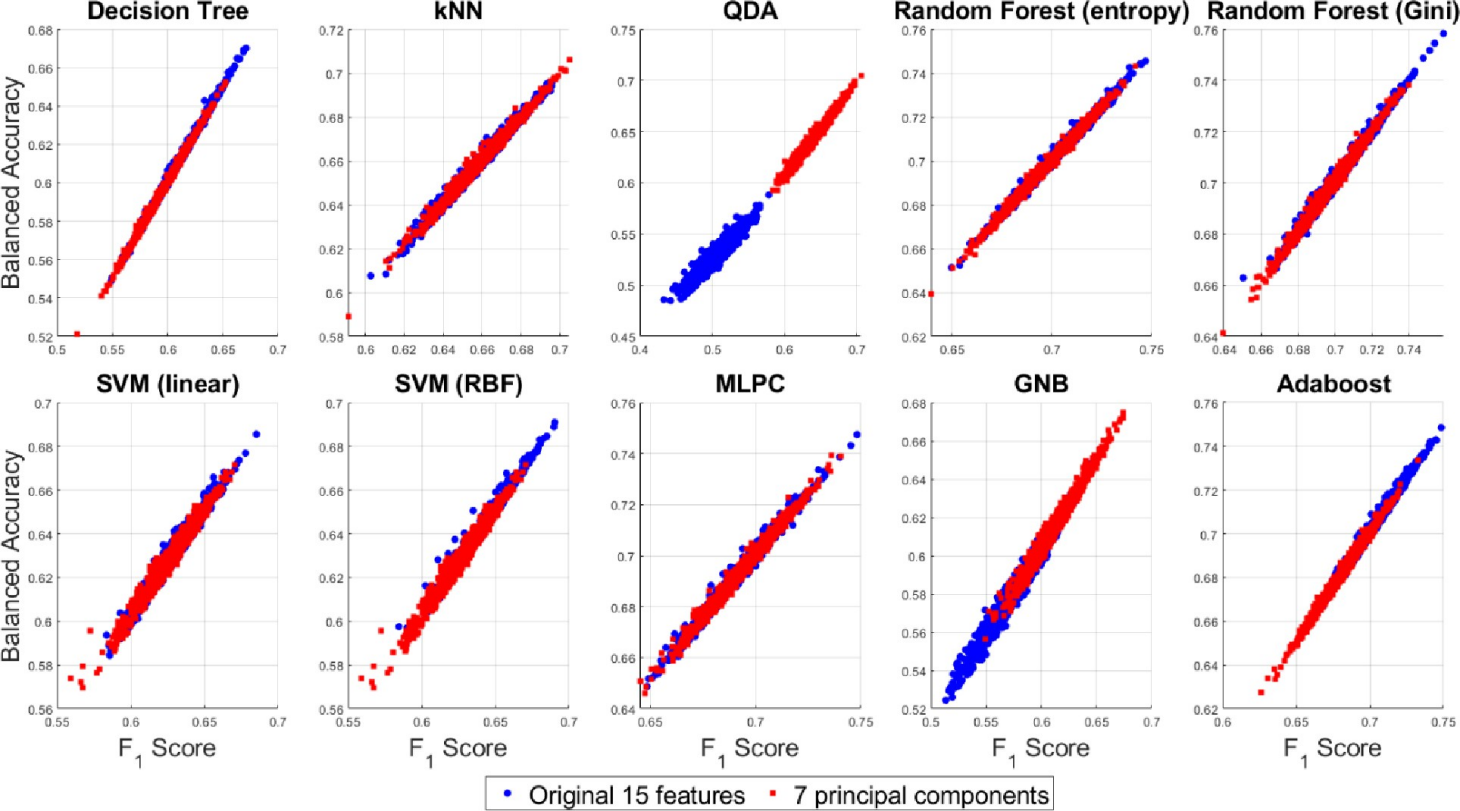
Scatterplots between the two performance measures–F_1_ score vs. balanced accuracy for the original 15D and 7D feature-based classification with different classifier families.

A 2D pairwise visualization of these three-performance metrics reveals more clearly which classifier and feature combinations (i.e. original vs. reduced features) are consistently giving better results as shown in Figs 15–17. These joint distributions show that the QDA and GNB yield better result up on dimensionality reduction via PCA, indicated by the centroid of the clusters in red circles. However, a slightly better result is observed using the random forest (Gini) and Adaboost classifiers using the original 15D feature space as compared to the reduced 7 PCs space, although there is a large degree of overlap between the performance metrics for both the 15D vs. 7D dimensions. All the three performance measures seem to be highly correlated for most of the classifiers except the QDA and GNB which shows wider spread in their performance metrics. Therefore, it can be summarized that all the three pairwise scatterplots in Figs 15–17 indicate a better performance in 7D PC space using the QDA and GNB classifiers. However, consistently the Adaboost classifier in the original 15D feature space clearly outperforms the other classifier-feature pairs irrespective of the chosen performance metrics.

### 3.5 Hypothesis testing between the reduced and the original feature space

Beside these intuitive visualizations to judge the classifiers performance in the previous section, multivariate hypothesis confirmatory tests can also be used. The aim here is to compare the mean-values or centroids of the 15D vs 7D classification metrics in terms of F_1_ score, MCC and balanced accuracy of the 1000 ensembles in the under-sapling. To achieve this, the one-way multivariate analysis of variance (MANOVA) hypothesis testing has been performed, considering that the same datapoints are not being used in all classifiers. The observations in the groups are random samples from the 15D and 7D, 1000 Monte Carlo classification runs and tests the null hypothesis that *multivariate means of each group are the same and any difference observed is due to random chance*. This MANOVA test in Table 4 confirms whether there is any significant difference in the multivariate distribution of these classification metrics for each of the 8 chosen classifier families (i.e. 10 classifiers). It is evident that all the classifiers except the MLPC fails to reject the null hypothesis that there is no significant difference in the mean values. This result can also be intuitively verified from the almost overlapping scatter diagrams in Figs 15–17 for the MLPC in the 15D and reduced 7D feature space. The test-statistics of the one-way MANOVA [63] viz. Wilk’s lambda test statistic (Λ), its transformation to an approximate *χ*2 distribution and the *p*-value are listed in Table 4 for each family of classifiers. However, this one-way MANOVA analysis makes three assumptions–

- firstly, sampled performance metrics for each group are closed to a multivariate normal distribution,
- secondly, the covariance matrix being almost same for each population, and
- thirdly, the observations or the three performance measures are mutually independent.

The third assumption is sometimes difficult to meet under a random Monte Carlo sampling framework which motivates us to investigate dependent MANOVA tests. However, a single MANOVA test for each classifier is superior than carrying out three different ANOVA tests for the three classification metrics since it reduces the chance of making type-I error [64]. Moreover, the performance metrics appear to be highly correlated as revealed from Figs 15–17. In previous works, classification algorithm performance measures have been mostly compared using *t*-test [65] and ANOVA [66]. although MANOVA has been previously used in combination with classifiers [67], but have not been used in the comparison of classifiers’ performance metrics as a joint distribution, as reported in this paper.

**Table 4:** Repeated measure MANOVA table combining all three performance measures of all 10 classifiers.

Within	Between	Statistic	Value	*F*-statistic	*R* ^2^	df1	df2	*p*-value
Constant	(Intercept)	Pillai (*V*)	0.904297	94475.54	0.904297	2	19997	0
Constant	(Intercept)	Wilks (Λ)	0.095703	94475.54	0.904297	2	19997	0
Constant	(Intercept)	Hotelling (*U*)	9.448972	94475.54	0.904297	2	19997	0
Constant	(Intercept)	Roy (Θ)	9.448972	94475.54	0.904297	2	19997	0
Constant	group	Pillai (*V*)	0.048526	509.9293	0.048526	2	19997	1.01×10^−216^
Constant	group	Wilks (Λ)	0.951474	509.9293	0.048526	2	19997	1.01×10^−216^
Constant	group	Hotelling (*U*)	0.051001	509.9293	0.048526	2	19997	1.01×10^−216^
Constant	group	Roy (Θ)	0.051001	509.9293	0.048526	2	19997	1.01×10^−216^

Following the one-way MANOVA hypothesis testing results as shown in Table 4, it may be argued that under the random under-sampling scheme to handle class imbalance, some of the datapoints may have been considered on multiple occasions between the 15D and reduced 7D datasets. Therefore, we here also report the repeated measure MANOVA to answer this question whether the dimensionality reduction has any consistent improvement on each classifier according to the three performance measures which are correlated amongst themselves, as revealed from Figs 15–17. Since there is a chance under random under-sampling, the same datapoints may be tested in the 15D original vs. 7D reduced PC spaces, the repeated measure hypothesis testing needs to be used as compared to the standard version. The Monte Carlo classification performance metrics are clubbed in two groups–original 15D vs. reduced 7D feature space for all the 10 classifiers and three classifier performance measure (each with 1000 samples), as required in the repeated measure MANOVA. The four standard statistics [64], used in MANOVA are–Wilk’s lambda (Λ), Pillai-Bartlett trace (*V*), Hotelling-Lawley trace (*U*), Roy’s maximum root statistic (Θ) and all of which reject the null hypothesis revealed from the low *p*-values in Table 4 which suggests that there is significant difference between the 15D vs. 7D classification scenarios. These four test-statistics can be calculated from the eigenvalues (*λ*_*i*_,*i* = 1,⋯,*s*) for each discriminant variates and *s* representing the number of variates. The Pillai-Bartlett trace (*V*) represents the sum of the proportion if explained variance on the discriminant functions:

$$V=\sum_{i=1}^{s}\lambda_{i}/\left(1+\lambda_{i}\right).$$ (6)

The Hotelling-Lawley trace (*U*) or Hotelling T^2^ represents the sum of the eigen-values for each variate as:

$$T=\sum_{i=1}^{s}\lambda_{i}.$$ (7)

The Wilk’s lambda (Λ) represents the product of the unexplained variance on each of the variates as:

$$\Lambda =\prod_{i=1}^{s}1/\left(1+\lambda_{i}\right).$$ (8)

Roy’s maximum root statistic (Θ) represents the proportion of explained variance to unexplained variance for the first discriminant as:

$$\Theta =\max(\lambda_{i}),\;i=1,\cdots ,s.$$ (9)

Amongst these four test statistics, the Hoteling T^2^ is suggested to be the most robust one, against the violation of multivariate normality condition for two group situation with equal sample size and lower number of dependent variable (3 in this case), as compared to the skewed sample size and many dependent variables [64]. However, previous research in [64, 68] have suggested that when samples sizes are equal, the Pillai-Bartlett trace is the most robust statistic against the violation of both multivariate normality and equal covariance matrix conditions. More details of these test statistics of repeated measure MANOVA can be found in [69].

**Fig 18:**
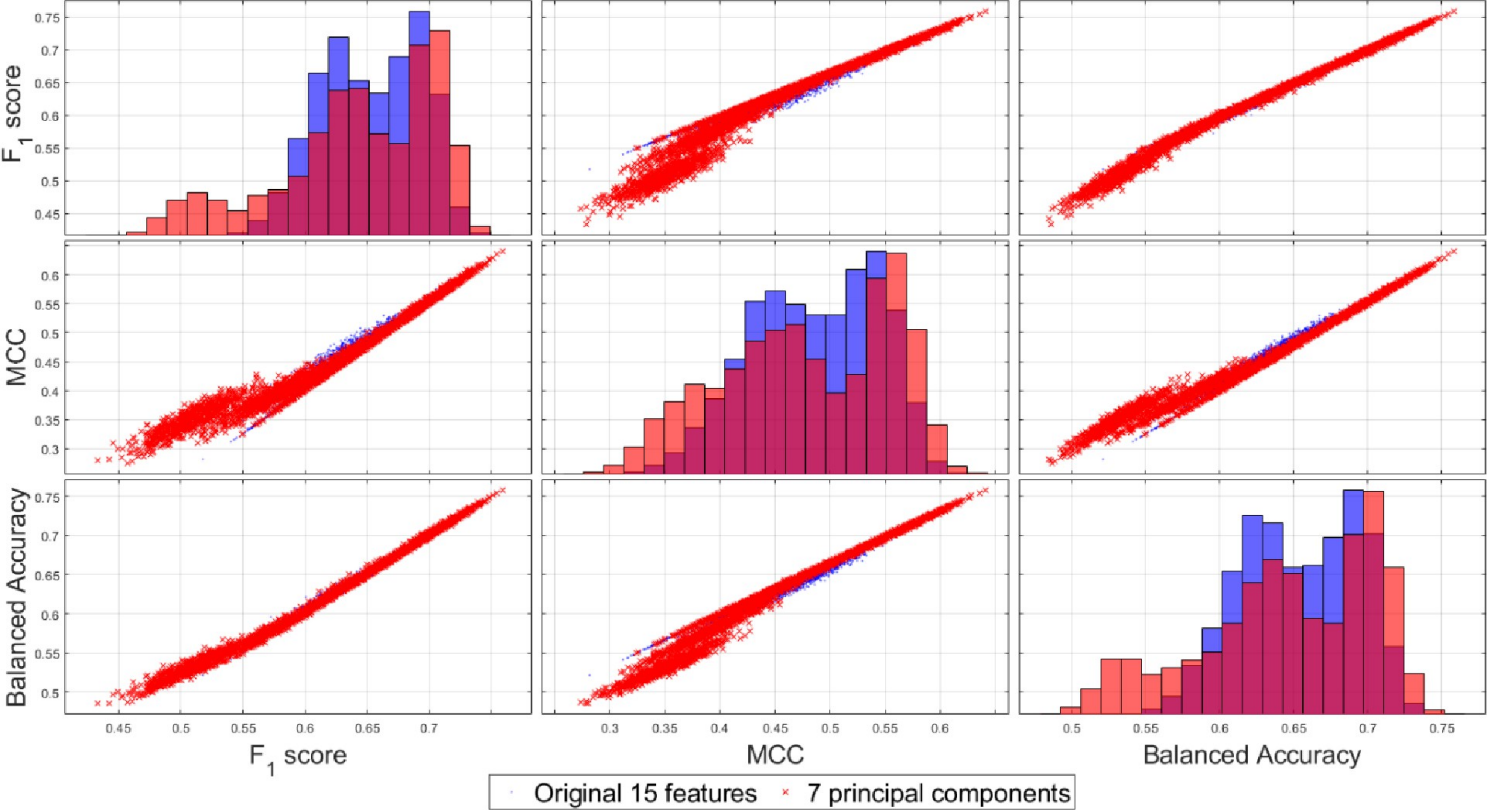
Groupwise scatter diagrams (off diagonals) and the 1D marginal distributions of the classification performance measures (along the principal diagonal), combining 1000 Monte Carlo under-sampling runs for all 10 classifiers. Blue dots represent samples from the original 15D classification, and the red crosses represent the reduced 7D classification results.

Next, we report the repeated measure group-wise MANOVA to compare these four test statistics for the 15D vs 7D classification results as shown in Table 4 with small *p*-values, indicating the rejection of the null hypothesis using all the four test-statistics. A similar group-wise repeated measure MANOVA can be used to compare these test-statistics for the original 15D vs. reduced 7D feature space-based classification results combing all the 10 family of classifiers which also shows rejection of the null hypothesis as revealed from Table 5. When combining all the 10 classifiers’ results, the group-wise analysis in Fig 18 shows the original 15D feature space has slightly higher probability of yielding better results in term of all the three classification performance metrics as compared to the 7D feature space, although they share a large overlapping region of performance using the three performance metrics more suitable for comparing unbalanced data classification problems.

**Table 5:** Groupwise repeated measure MANOVA table combining all three performance measures of all 10 classifiers in the original 15D feature space vs. the 7D reduced feature space.

Within	Between	Statistic	Value	*F*-statistic	*R* ^2^	df1	df2	*p*-value
Constant	group = 7D	Pillai (*V*)	0.975147	392313.5	0.975147	2	19997	0
Constant	group = 7D	Wilks (Λ)	0.024853	392313.5	0.975147	2	19997	0
Constant	group = 7D	Hotelling (*U*)	39.23723	392313.5	0.975147	2	19997	0
Constant	group = 7D	Roy (Θ)	39.23723	392313.5	0.975147	2	19997	0
Constant	group = 15D	Pillai (*V*)	0.974384	380328.7	0.974384	2	19997	0
Constant	group = 15D	Wilks (Λ)	0.025616	380328.7	0.974384	2	19997	0
Constant	group = 15D	Hotelling (*U*)	38.03857	380328.7	0.974384	2	19997	0
Constant	group = 15D	Roy (Θ)	38.03857	380328.7	0.974384	2	19997	0

In order to summarize, the thorough comparison between the classifier families, performance metrics for the plant signal classification problem, we also show comparison of the current research with the other similar works on chemical stimuli classification. In particular, we compare the statistical features, species and classifiers in Table 6 where we clearly show the comparison using multiple performance metrics on a comparable dataset and using a greater number of classifier families. Table 6 shows that the complexity of the current exploration is much higher as compared to the previous works in terms of number of features, classification metrics, and comparison of classifiers using wide variety of hypothesis tests. There is no previous work that reports statistical analysis of the classification results using multiple classification metrics using plant electro-physiological data under different chemical stress.

**Table 6:** Comparison of contemporary chemical stress classification methods from plant electrophysiological data.

Reference	Species used for data collection	No. of data blocks / time series used for data analysis	No. of stimuli	No. of features used	Classifiers used	Metric used	Type of signal used for analysis
Chatterjee *et al*. [3]	Tomato, Cucumber	3801 data blocks (1000 samples per block)	4	11	5 (LDA, QDA, Diaglinear, Diagquadratic, Mahalanobis)	Accuracy	Raw
Chatterjee *et al*. [4]	Tomato, Cucumber, Cabbage	38186 data blocks (1024 samples per block)	3	15	5 (LDA, QDA, Diaglinear, Diagquadratic, Mahalanobis)	Balanced accuracy	Raw and filtered
Chatterjee *et al*. [6]	Tomato, Cucumber, Cabbage	411 time series (no segmentation or blocks)	3	Coefficients from 4 curve fitting methods—Polynomial (9 degrees), Gaussian (4 degrees), Fourier (4 degrees), Exponential (2 degrees)	5 (LDA, QDA, Diaglinear, Diagquadratic, Mahalanobis)	Sensitivity, Specificity, Accuracy	Raw
This paper	Tomato, Cabbage	37834 data blocks (1024 samples per block)	3	15	8 (decision tree, kNN, QDA, random forest, SVM, MLPC, GNB, Adaboost)	Balanced accuracy, F_1_-score, Matthews correlation coefficient	Filtered

## 4. Conclusions and future scope of research

Plant signal classification is an important challenge in precision agriculture and environmental monitoring. However due to high complexity of such plant electrical signals, this paper at first extracts explainable higher order statistical features in a non-overlapping sliding window fashion. The three environmental chemical stimuli–sulfuric acid, ozone and sodium chloride yields plant electrical response datasets of different length making the problem highly unbalanced. Such an unbalanced classification problem becomes difficult to be tackled by standard machine learning algorithms and were circumvented by a Monte Carlo under-sampling approach to create ensemble of confusion matrices. We compare the predictive accuracies and three other performance metrics and their distributions across 1000 ensembles of random under-sampling, which are especially relevant for unbalanced multi-class problems. Our exploratory data analysis shows that the Adaboost classifier in the original 15D feature space yields the highest average classification performance. Moreover, the PCA versions do not perform well in this setting due to high complexity of the dataset and the use of nonlinear features in this classification problem. When combining all the classifiers, the PCA do not give significant improvement as revealed from the repeated measure MANOVA tests. However, while considering individually, the Adaboost followed by random forest (gini) performs the best in terms of all the three-classification metrics in the reduced 7D space of PCs. Future work can be directed towards applying unsupervised learning methods on similar plant electrophysiological monitoring datasets for mining patterns and statistical modelling. Unlike the current paper, no previous work makes a systematic comparison between the principal component vs. statistical feature-based classification and quantified the differences using multivariate hypothesis tests utilizing multiple classification metrics obtained from the Monte Carlo cross-validation runs. The main contribution of this paper is the thorough benchmarking of the plant electrophysiological response data classification with a family of classifiers using multiple performance metrics on the high vs. reduced dimensional unbalanced data. We also quantify and compare the significance of these differences between the effect of dimensionality and classifier family through MANOVA tests.

The electrical signal response of the plants has been shown to contain invaluable information about the various factors (stimuli) affecting them. This information can be used as a means of sensing the environment in which the plants are growing. Since plants are found in abundance naturally, they can be used to provide environmental information about a large geographical area. The possibility of such large-scale geographical monitoring seems immense since when such electrical signals are extracted and analyzed, crucial and timely information may be found. A successful analysis of such plant electrical signals will assist the ambition of plants being used as a living, multiple stimuli environmental biosensor which are capable of generating estimates of chemical pollutants in the environment like ozone close to more expensive chemical sensors [70]. However, such a plant-based biosensor development needs to consider the electronic hardware implementation challenges. This paper does not address the question of feasibility of the data analysis for real-time implementation for streaming plant time-series data. Because in a real field data monitoring scenario, the classification system may need more involved research regarding the speed, data storage and memory requirements of the electronic hardware which is beyond the scope of this paper.

Apart from a macro scale detection, utilization of such electrical signal response in plants may also provide timely information about common infections such as certain types of fungus or pest’s infestations. This type of electrical signal responses, when appropriately analyzed–using artificial intelligence and machine learning techniques, will be able to alarm the farmers about the infection or diseases the plant is suffering from at the early onset. In addition to knowing when a plant has been infected, we may also know what type of infection or pollutant it is, thereby inducing a timely cure or precaution which may save the farmers lots of money. Such an intelligent system will contribute towards further modernizing current practices in the precision agriculture methods.
